# Supplementary material for: Fate-mapping post-hypoxic tumor cells reveals a ROS-resistant phenotype that promotes metastasis
Source: Nat Commun. 2019 Oct 24;10:4862. doi: 10.1038/s41467-019-12412-1 (PMC6813355; doi:10.1038/s41467-019-12412-1)
Supplement: Supplementary file 1 — Supplementary Information [file 41467_2019_12412_MOESM1_ESM.pdf]

Fate-mapping post-hypoxic tumor cells reveals a ROS-resistant phenotype that promotes metastasis

Godet *et al.*

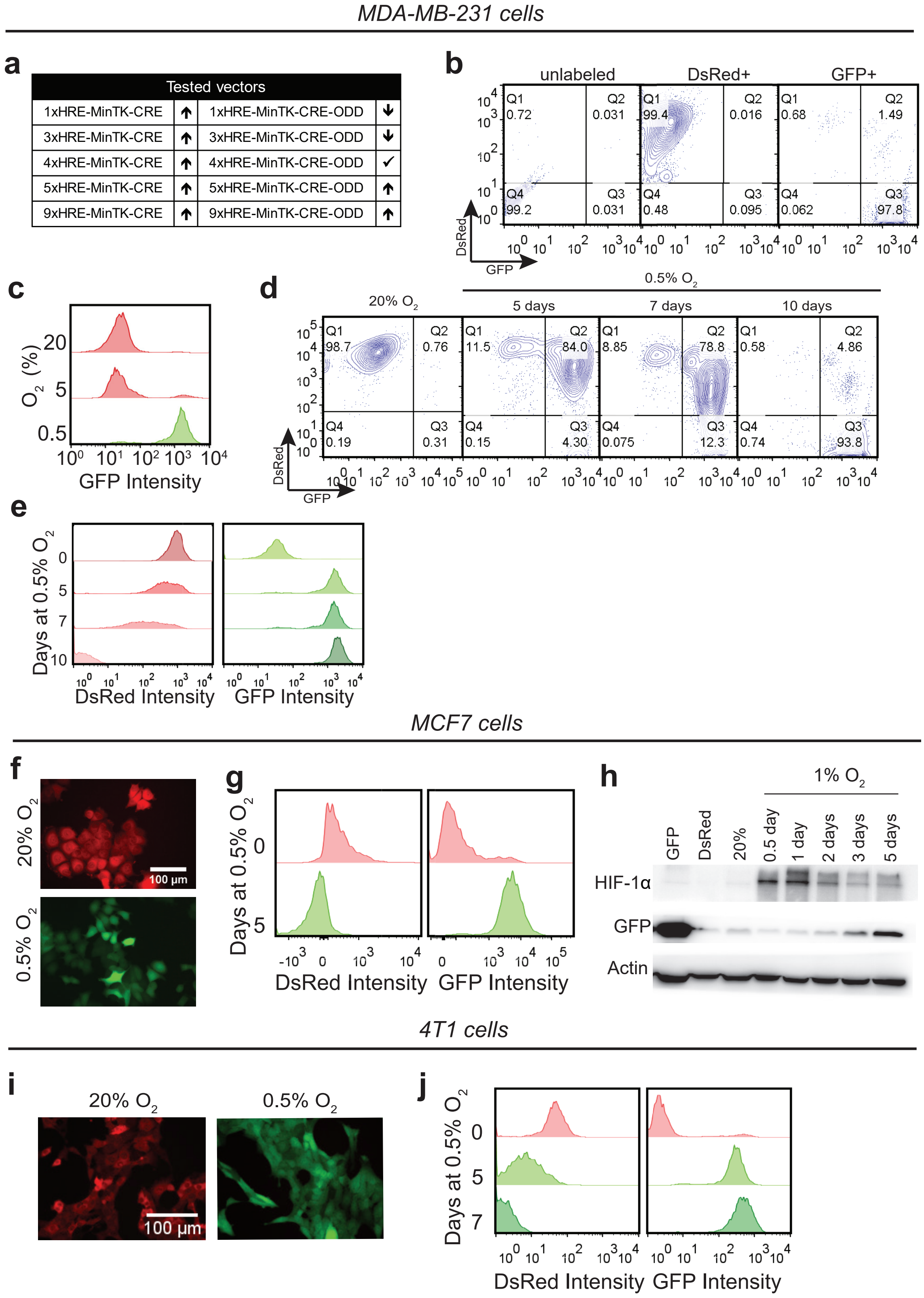

Supplementary Figure 1. Establishing a hypoxia fate-mapping system in breast cancer cell lines. a, Multiple designs for vector 2 were tested to achieve a tightly-controlled response to <0.5% O_2. Some designs resulted in no GFP expression (arrow down) even under 0.5% O_2, whereas GFP was detected at >0.5% O_2 in alternate designs that contained more than 5 HREs and without the addition of the ODD domain (arrow up). **b,** Contour plots displaying the gating strategy for flow cytometry analysis of DsRed and GFP expression in MDA-MB-231 hypoxia fate-mapping cells. **c,** Histogram plots of DsRed or GFP intensity as measured by flow cytometry of MDA-MB-231 fate-mapping cells exposed to 20, 5 or 0.5% O_2. **d,** Contour plots of flow cytometry data for MDA-MB-231 fate-mapping cells exposed to 20% or 0.5% O_2 conditions for 5, 7 or 10 days. **e,** Histogram plots corresponding to contour plots in (**d**) of DsRed or GFP intensity as measured by flow cytometry. **f-g,** Fluorescent images (**f**) and representative histogram plots from flow cytometry analysis (**g**) of MCF7 hypoxia fate-mapping cells exposed to 20% or 0.5% O_2 for 7 days. **h,** Immunoblot of MCF7 hypoxia fate-mapping cells cultured under 20% or 1% O_2 for 0.5, 1, 2 or 3 days. DsRed or GFP only cell lines were used as controls. **i-j,** Fluorescent images (**i**) and representative histogram plots from flow cytometry analysis (**j**) of 4T1 hypoxia fate-mapping cells exposed to 20% or 0.5% O_2 for 7 days.

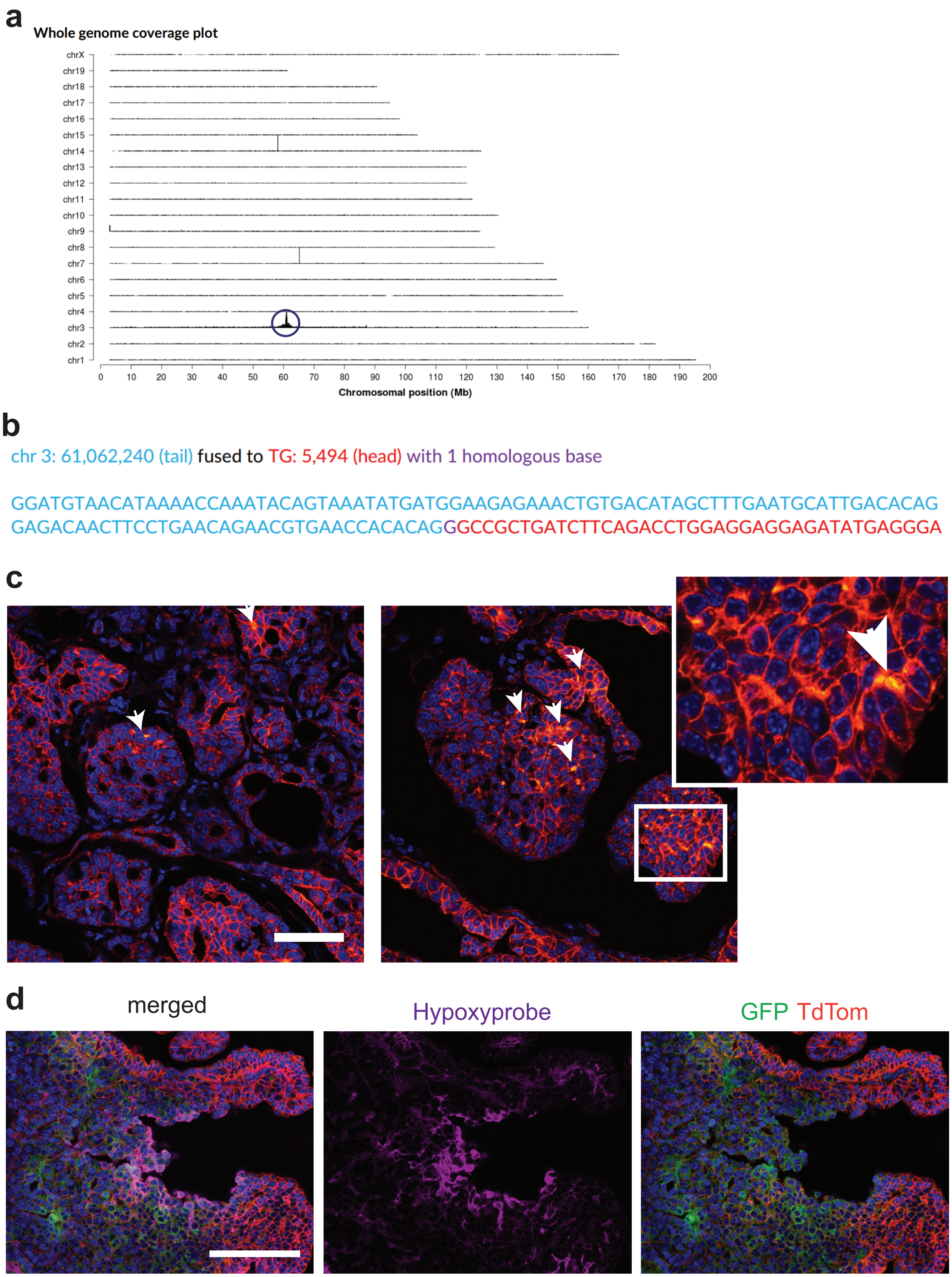

Supplementary Figure 2. Establishing a hypoxia fate-mapping triple-transgenic mouse model. a, TLA sequence coverage across the mouse genome using primer set 2 (Supplementary Table 2). The chromosome number is indicated on the y-axis and the chromosomal position on the x-axis. Similar results were obtained with primer set 1 (Supplementary Table 2). The 4xHRE-CREODD transgene integrated at chromosome 3 position 61,062,240. **b,** Nucleotide sequence of the transgene integration site. **c,** Fluorescent images of ductal carcinoma *in situ* (DCIS) during MMTV-PyMT tumor progression. Arrows point to regions of hypoxia developing in DCIS lesions. Scale bar: 50 µm. Inset shows an enlarged view. **d,** Fluorescent image of a triple-transgenic mouse tumor that was harvested and fixed in 4% PFA. After freezing and cryo-sectioning, the tumor was stained for Hypoxyprobe™ (purple). Scale bar: 100 µm.

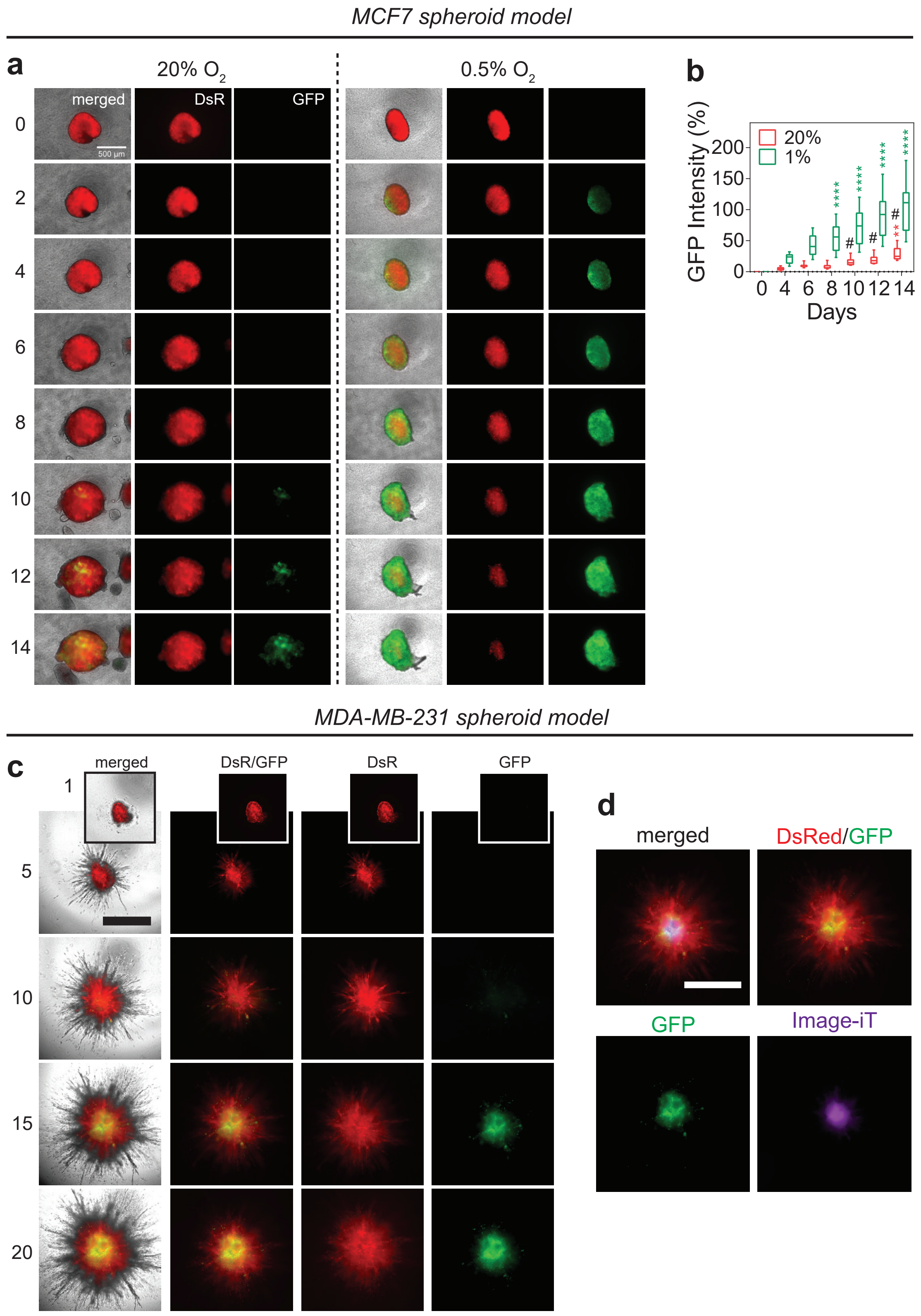

Supplementary Figure 3. Hypoxia fate-mapping system in a spheroid model. a, Spheroids consisting of MCF7 hypoxia fate-mapping cells were fully embedded in a 2 mg/ml collagen gel and cultured for 14 days under 20% or 1% O_2. **b,** GFP intensity at each time point was normalized by the GFP intensity measured on day 0 (background) for spheroids cultured under 20% or 1% O_2 (mean ± SEM, N=2, n=16); **** $P<0.0001$ versus 20% day 4 (red) or 1% day 4 (green), and #$P<0.0001$ for 20% versus 1% (Two-way ANOVA with Bonferroni post-test). The box extends from the 25th to 75th percentiles, the median is marked by the vertical line inside the box, and the whiskers represent the minimum and maximum points. **c,** Fluorescent images of MDA-MB-231 spheroids embedded in 2 mg/ml collagen gels that were cultured under 20% O_2 and monitored over a 20-day time course. Scale bar: 1mm. **d,** Fluorescent images of an MDA-MB-231 spheroid treated with Image-iT™ hypoxia reagent for 3 h at 37°C to detect cells that are hypoxic at the time of imaging.

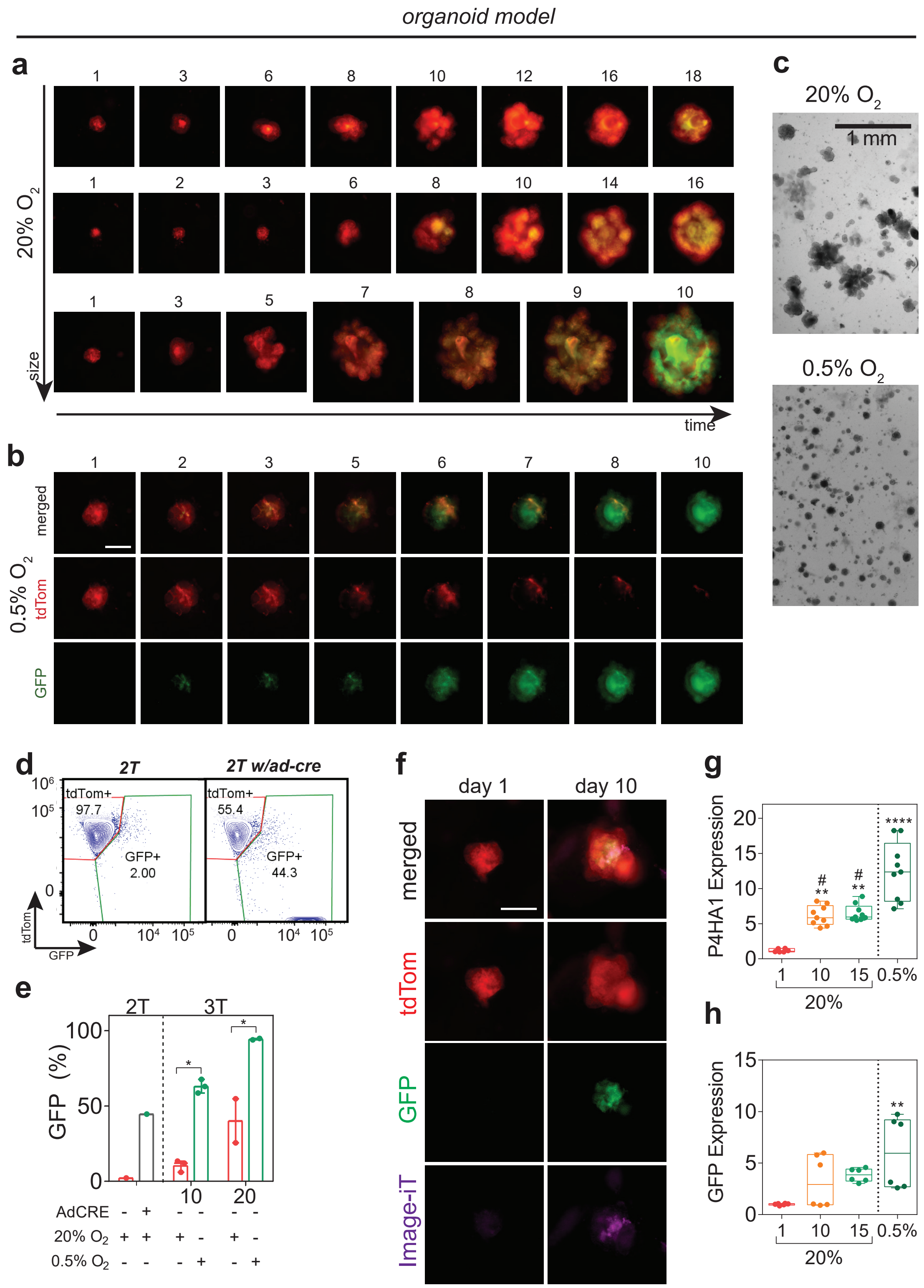

Supplementary Figure 4. Hypoxia fate-mapping system in an organoid model. a, Fluorescent images of mammary tumor organoids derived from triple-transgenic (3T) mice that were cultured under 20% O_2 and monitored for 10 to 18 days. Scale bar: 200 µm. **b,** Fluorescent images of 3T organoids that were embedded in Matrigel and exposed to 0.5% O_2 during a 10-day time period. Scale bar: 100 µm. **c,** Bright-field images of 3T organoids that were embedded in Matrigel and cultured under 20% or 0.5% O_2 for 10 days. **d,** Gating strategy for flow cytometry analysis of tdTom and GFP expression in organoids. Organoids derived from double-transgenic mice (2T) with the mT/mG and MMTV-PyMT transgenes were used as tdTom+/GFP control. The same organoids treated with Ad-cre were used as the GFP+ control. **e,** Percentage of GFP+ cells in organoids analyzed by flow cytometry (mean ± SEM, N=2-3, n>200); **** P<0.0001 versus 20% (Two-way ANOVA with Bonferroni post-test). **f,** Fluorescent images of 3T organoids that were embedded in Matrigel (3D) and cultured under 20% O_2 for 1 or 10 days. Organoids were incubated with Image-iT™ for 3 h at 37°C to mark hypoxic regions. Scale bar: 100 µm. **g-h,** Relative expression of P4HA1 (**g**) and GFP (**h**) mRNA levels were measured by qPCR in 3T organoids cultured for 1, 10 or 15 days under 20% or under 0.5% O_2 for 10 days (mean ± SEM, N=2-3, n=3); **** P<0.0001 versus Day 1 and # P<0.0001 versus 0.5% O_2 (one-way ANOVA with Bonferroni post-test). The box extends from the 25th to 75th percentiles, the median the vertical line inside the box, and the whiskers represent the minimum and maximum points. Primer sequences are available in Supplementary Table 1.

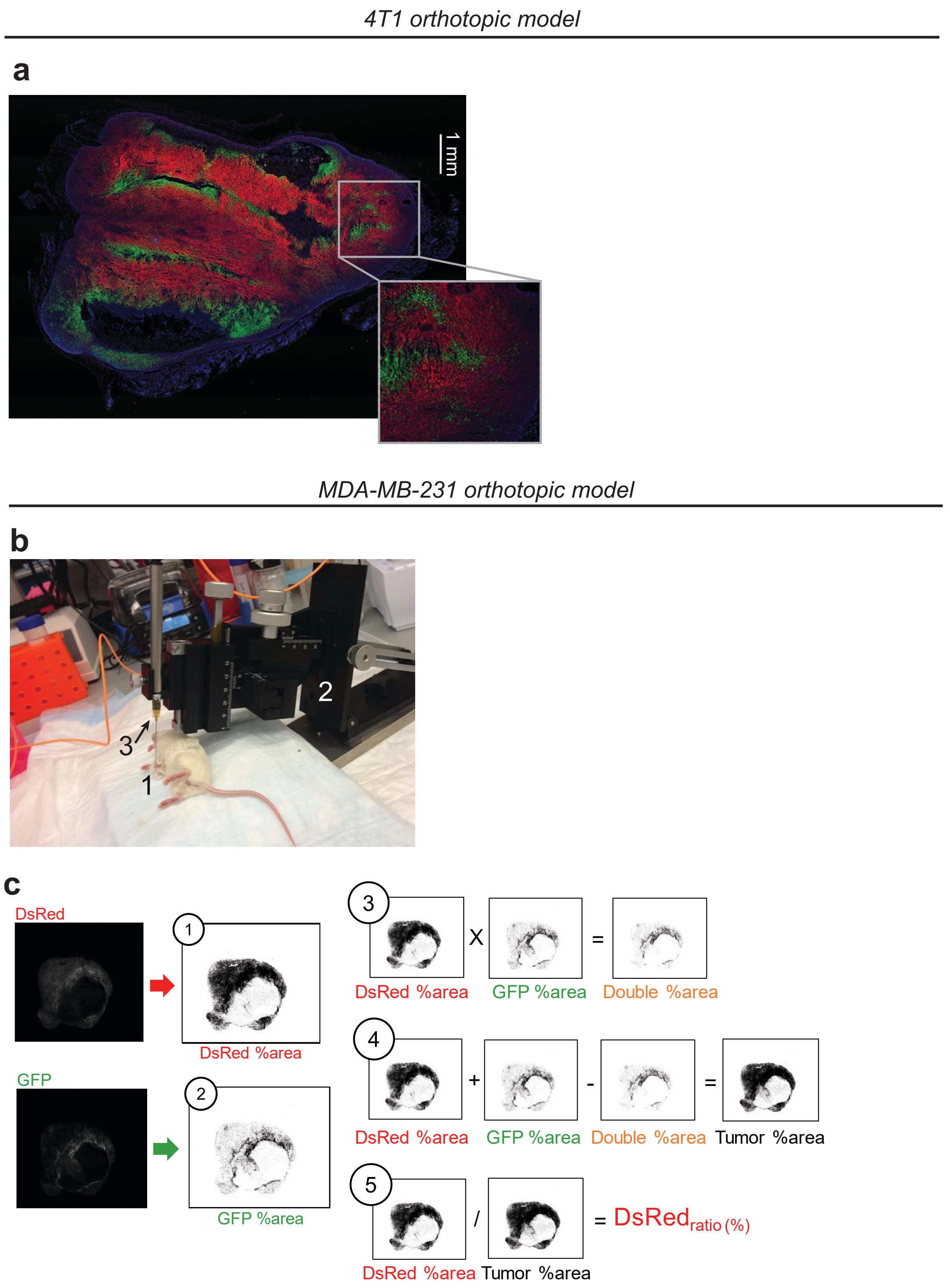

Supplementary Figure 5. Implementing a hypoxia fate-mapping system in breast cancer models. a, Fluorescent image of DsRed and GFP expression with DAPI staining of a full cross-section of an orthotopic mammary tumor in a BALB/c mouse derived from 4T1 hypoxia fate-mapping cells. Inset shows GFP+ single cells far from the hypoxic region. **b,** O_2 measurements were conducted in breast tumors of living mice with a fixed-needle microsensor (1). The microsensor was positioned into the tumor using a micrometer (2) mounted to a stand and connected to a laptop. O_2 concentrations were obtained starting at the midpoint of the tumor (3) and the micrometer was used to retract the sensor to capture measurements at 0.5 mm intervals until the sensor reached the tumor edge. **c,** Full MDA-MB-231 tumor cross-sections were imaged in tiles according to tumor size (up to 15x15 images) and linearly stitched. Using ImageJ, a threshold was manually applied to each fluorescent channel and converted into a binarized image (1, 2). Regions expressing both DsRed and GFP (double) were determined by the multiplication of the DsRed and GFP binarized images using the Image Calculator plugin (ImageJ) (3). The tumor area was estimated by the sum of DsRed and GFP binarized images and subtraction the double binarized area calculated using the Image Calculator plugin (4). Using individual binarized images divided by total area, we determined the ratio (%) of DsRed, GFP and double (DsRed/GFP) positive areas (5).

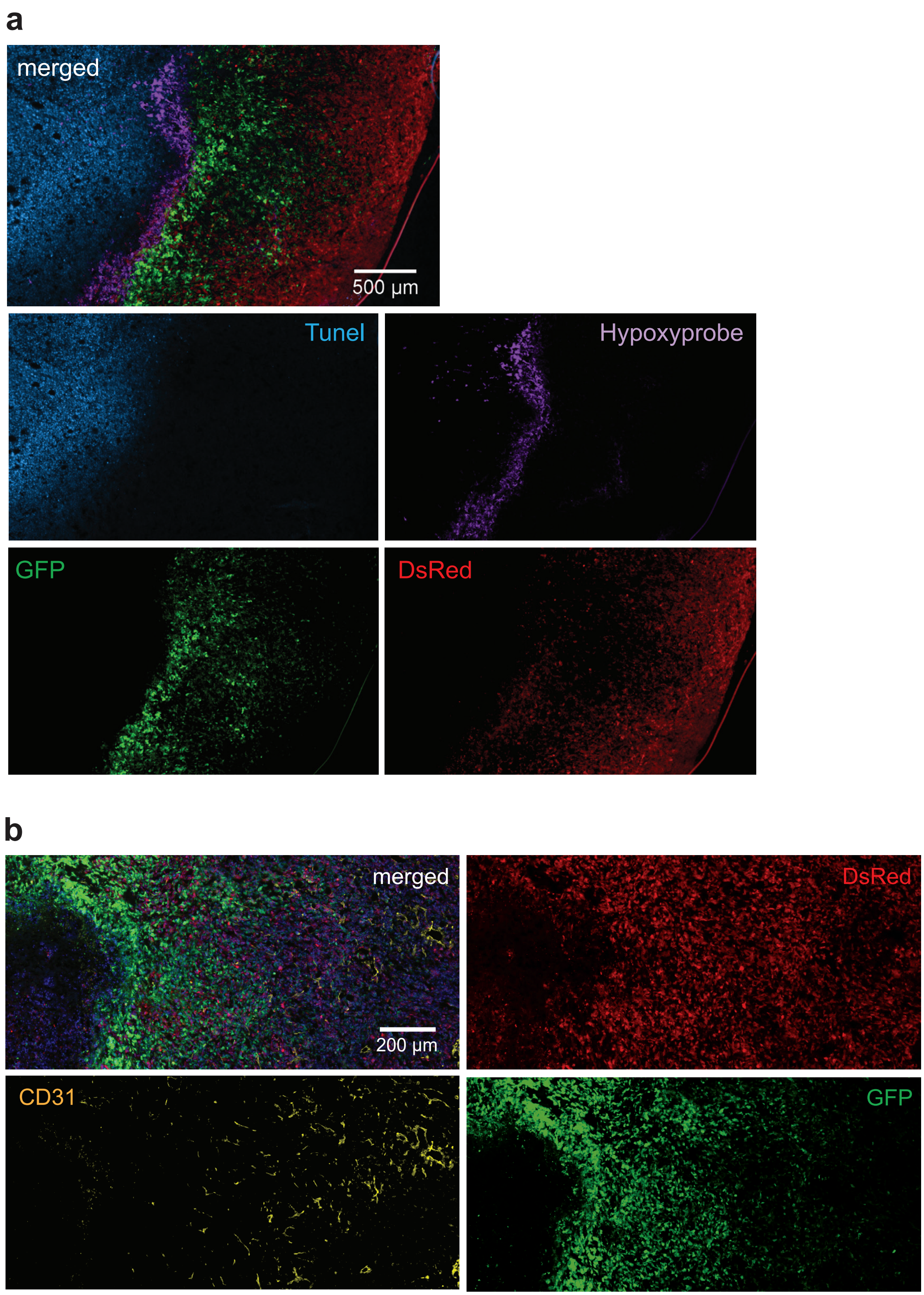

Supplementary Figure 6. Characterizing the hypoxia fate-mapping MDA-MB-231 orthotopic model. a, Fluorescent images of DsRed and GFP expression and TUNEL (blue) and Hypoxyprobe™ (purple) labeling. **b,** Fluorescent images of DsRed and GFP expression, and CD31 (yellow) immunofluorescent labeling to detect endothelial cells lining blood vessels.

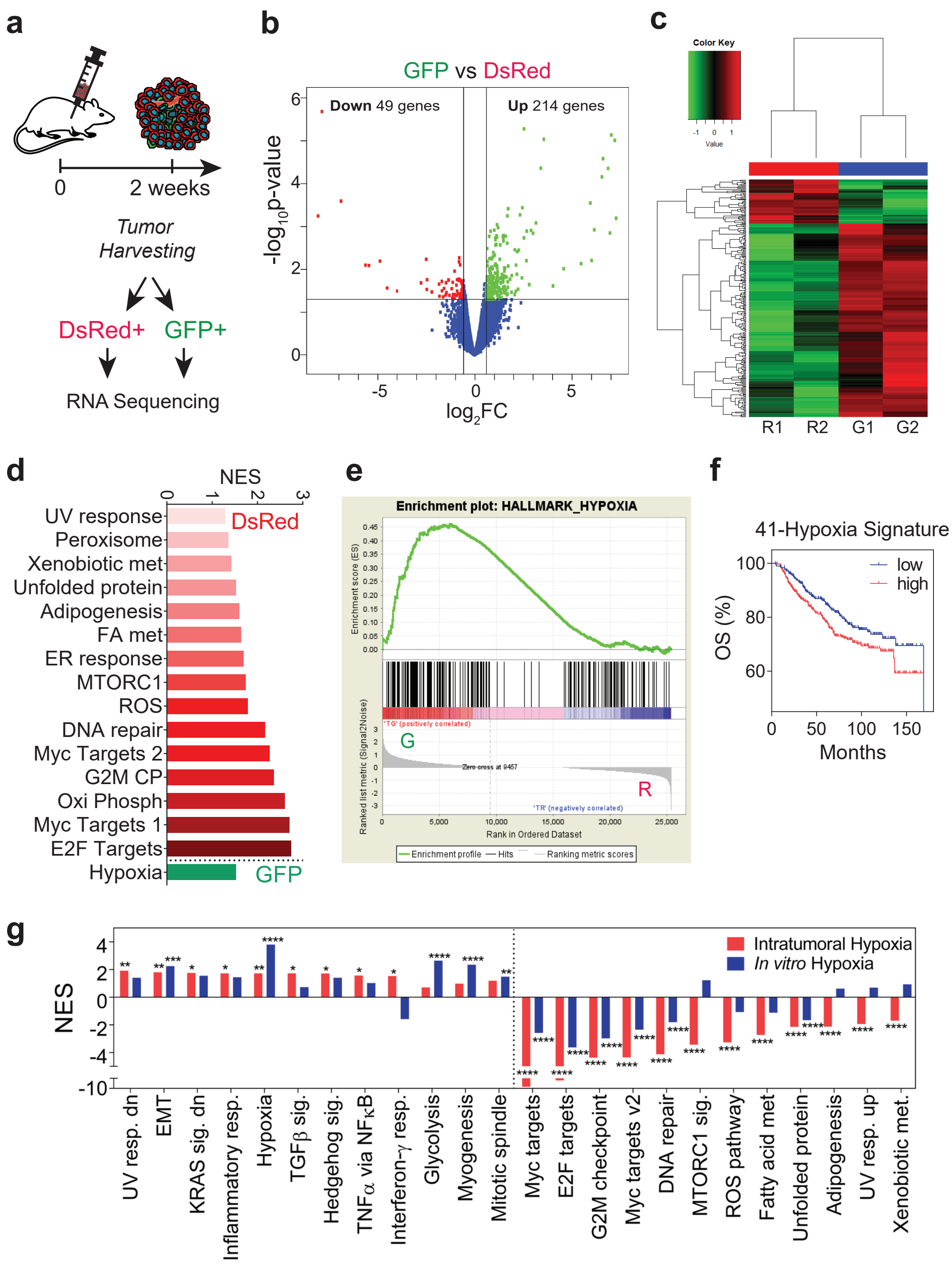

Supplementary Figure 7. RNA sequencing of DsRed+ and GFP+ sorted tumor cells and survival analysis. a, Tumors derived from hypoxia fate-mapping MDA-MB-231 cells were harvested 2 weeks after implantation, mechanically and enzymatically digested, and sorted into DsRed+/GFP- or GFP+ populations directly into Trizol (N=2) to prepare RNA. **b,** Volcano plot of gene expression from RNA sequencing analysis comparing GFP+ versus DsRed+ tumor cells. Red – down-regulated genes; Green – up-regula- ted genes (-1.5≥FC≥1.5) and p-value<0.05. **c,** Heatmap of gene expression from RNA sequencing analysis which compares RNA expression in GFP+ (G1 and G2) to DsRed+ tumor cells (R1 and R2). **d,** Normalized enrichment scores (nominal p-value ≤ 0.05) derived from (Gene Set Enrichment Analysis) GSEA against Hallmark gene sets of DsRed+ and GFP+ cancer cells purified from resected tumors. **e,** GSEA enrichment analysis for hallmark Hypoxia upregulated in class GFP+ (G). Normalized Enrichment score (NES) = 1.54. Nominal p-value = 0.0012. **f,** Kaplan–Meier analysis of overall survival (OS) of breast cancer patients stratified by high or low expression of the 41-gene signature derived from the overlap of intratumoral and *in vitro* hypoxia, according the median expression of the gene set. **g,** Normalized enrichment scores (**** nominal P< 0.0001) derived from pre-ranked (Gene Set Enrichment Analysis) GSEA against Hallmark gene sets. 'Intratumoral' and '*In vitro*' hypoxia matched gene lists were ranked according to the hypoxic induction (fold-change) in each condition.

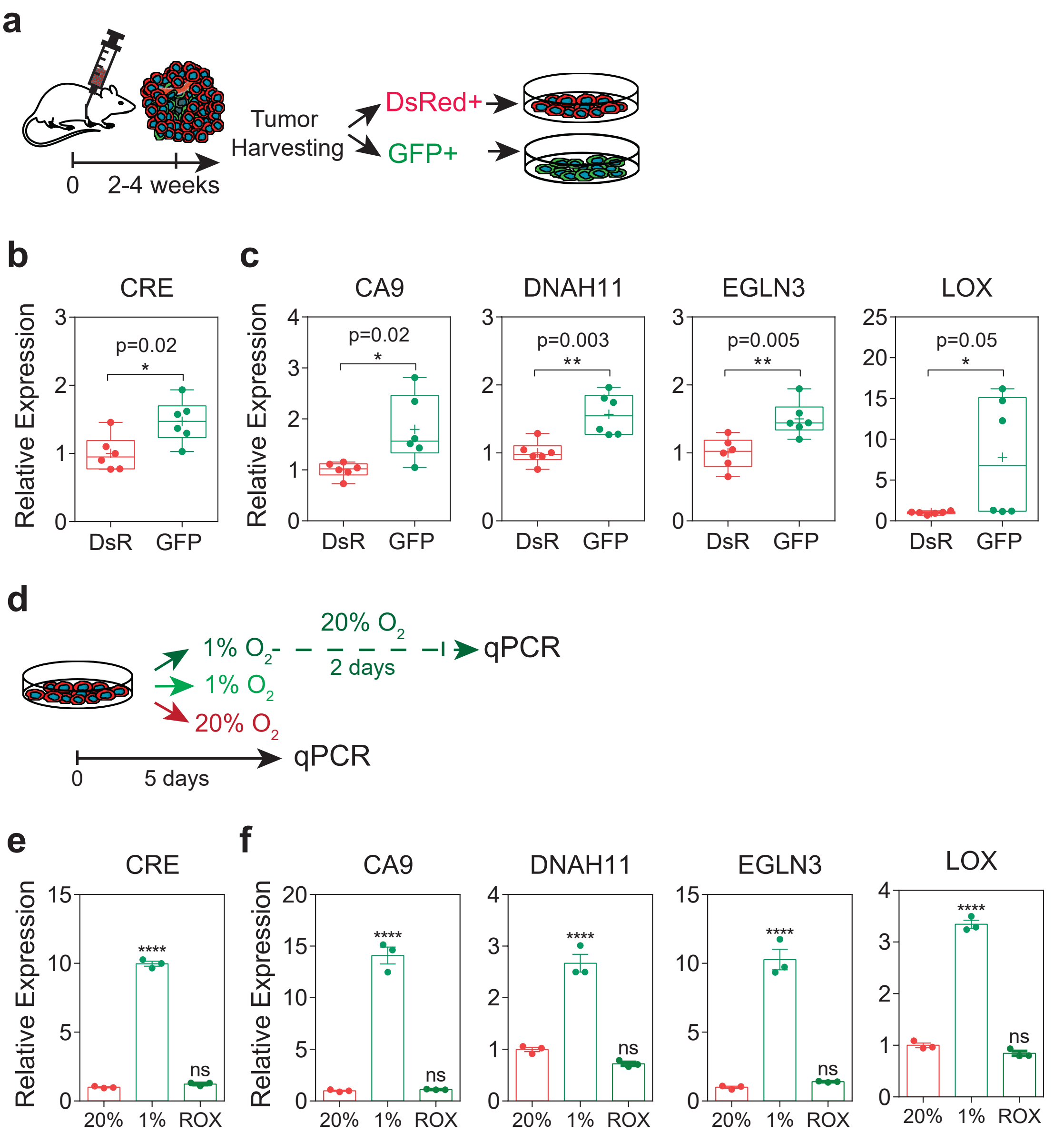

Supplementary Figure 8. Comparing reoxygenation effects in gene expression after *in vivo* or *in vitro* hypoxia. a, Cells were recovered from hypoxia fate-mapping MDA-MB-231 tumors by FAC-sorting directly into cell culture media (DsRed+/GFP- or GFP+). Cells were kept in culture for ~20 doublings (5-6 passages) and gene expression was tested using qPCR. **b,** Relative expression of CRE mRNA levels measured by qPCR in fate-mapping tumor-derived MDA-MB-231 cells (mean ± SEM, N=2, n=3); **** P<0.0001 GFP versus DsRed (two-tailed t-test). **c,** Relative expression of mRNA levels of selected genes present in the Venn diagram overlap of *in vivo* and *in vitro* hypoxia. CA9 (Carbonic Anhydrase 9), DNAH11 (Dynein Axonemal Heavy Chain 11), EGLN3 (Egl-9 Family Hypoxia Inducible Factor 3) and LOX (Lysyl Oxidase) were measured by qPCR in fate-mapping tumor-derived MDA-MB-231 cells (DsRed or GFP) (mean ± SEM, N=2, n=3); **** P<0.0001 GFP versus DsRed (two-tailed t-test). The box extends from the 25th to 75th percentiles, the median is marked by the vertical line inside the box, and the whiskers represent the minimum and maximum points. **d,** MDA-MB-231 cells were exposed to 1% O_2 for 5 days and reoxygenated (ROX) at 20% O_2 for 48 hours. **e,** Relative expression of CRE mRNA levels measured by qPCR in MDA-MB-231 cells (mean ± SEM, n=3); **** P<0.0001 versus 20% (one-way ANOVA with Bonferroni post-test). **f,** Relative expression of mRNA levels of selected genes present in the Venn diagram overlap of *in vivo* and *in vitro* hypoxia. CA9, DNAH11, EGLN3 and LOX were measured by qPCR in MDA-MB-231 cells (mean ± SEM, n=3); **** P<0.0001 versus 20% (one-way ANOVA with Bonferroni post-test). Primer sequences are available in Supplementary Table 1.

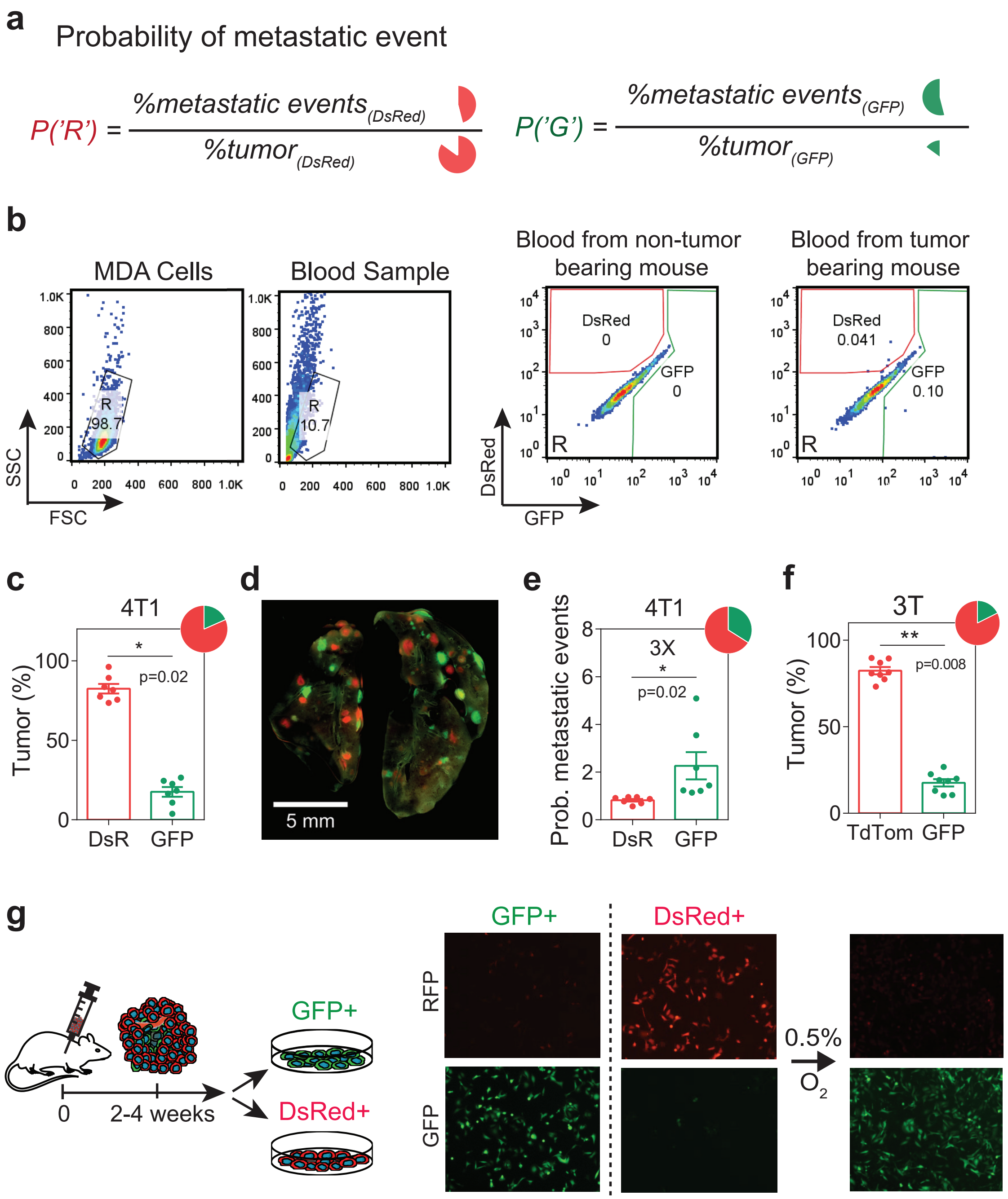

Supplementary Figure 9. Post-hypoxic cells have enhanced metastatic potential. a, The probability of having a GFP+ metastatic event compared to a DsRed+ event was calculated by dividing the ratio of DsRed+ or GFP+ cells at the metastatic site by the ratio of DsRed+ or GFP+ cells present in the matched tumor and then normalizing to DsRed+. **b,** Dot plots displaying the gating strategy for flow cytometry analysis of DsRed and GFP expression in MDA-MB-231 hypoxia fate-mapping CTCs. Cancer cell population was gated (R) in SSC versus FSC based on MDA-MB-231 cells. Gated region (R) is analyzed in terms of DsRed vs GFP. **c,** Ratio of DsRed+ and GFP+ cells in hypoxia fate-mapping 4T1 tumors in NSG mice resected 12 days post-implantation (mean ± SEM, N=7); **** P<0.0001 GFP versus DsRed (two-tailed t-test). **d-e,** Lungs were harvested 2 weeks after tumor removal to be imaged for DsRed and GFP (**d**) and probability of metastatic events in the lung was obtained by normalizing number of detected colonies by percentage of cells present in the tumor using image analysis (**e**). **f,** Ratio of tdTom+ and GFP+ cells in the tumors of hypoxia fate-mapping triple-transgenic mice (mean ± SEM, N=8); **** P<0.0001 GFP versus tdTom (two-tailed t-test). **g,** Cells recovered from a hypoxia fate-mapping MDA-MB-231 tumors were sorted directly into media (DsRed+/GFP- or GFP+) and maintained in culture. DsRed+ cells were exposed to 0.5% O_2 for 7 days and imaged for DsRed and GFP to show that they retain the ability to activate GFP expression under hypoxia.

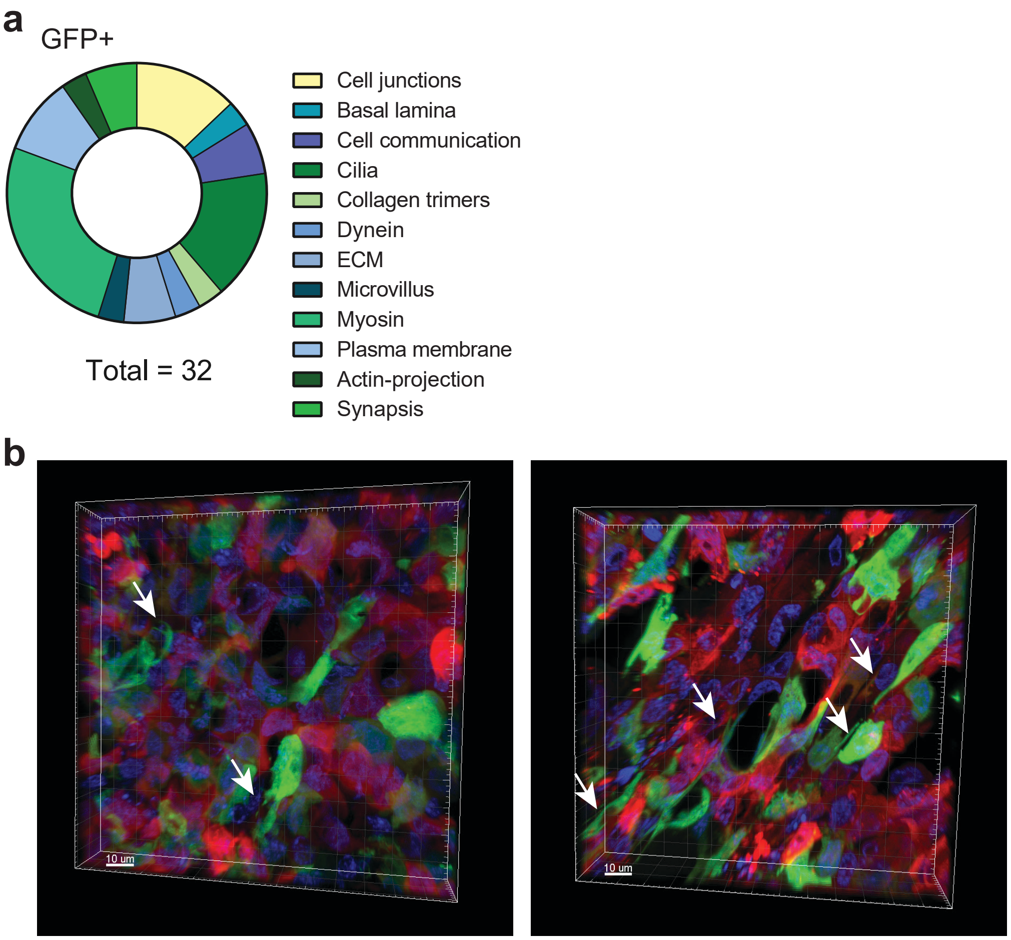

Supplementary Figure 10. Post-hypoxic cells have enhanced invasion. a, Gene Set Enrichment Analysis (GSEA) against GO – Cellular Component gene sets of GFP+ versus DsRed+ cancer cells isolated from harvested tumors. Enriched pathways with nominal p-value ≤ 0.05 were grouped into categories. **b,** 3D-reconstruction of cancer cells at the periphery of a tumor section stained with DAPI and mounted on a slide. Arrows indicate protrusions in GFP+ cells.

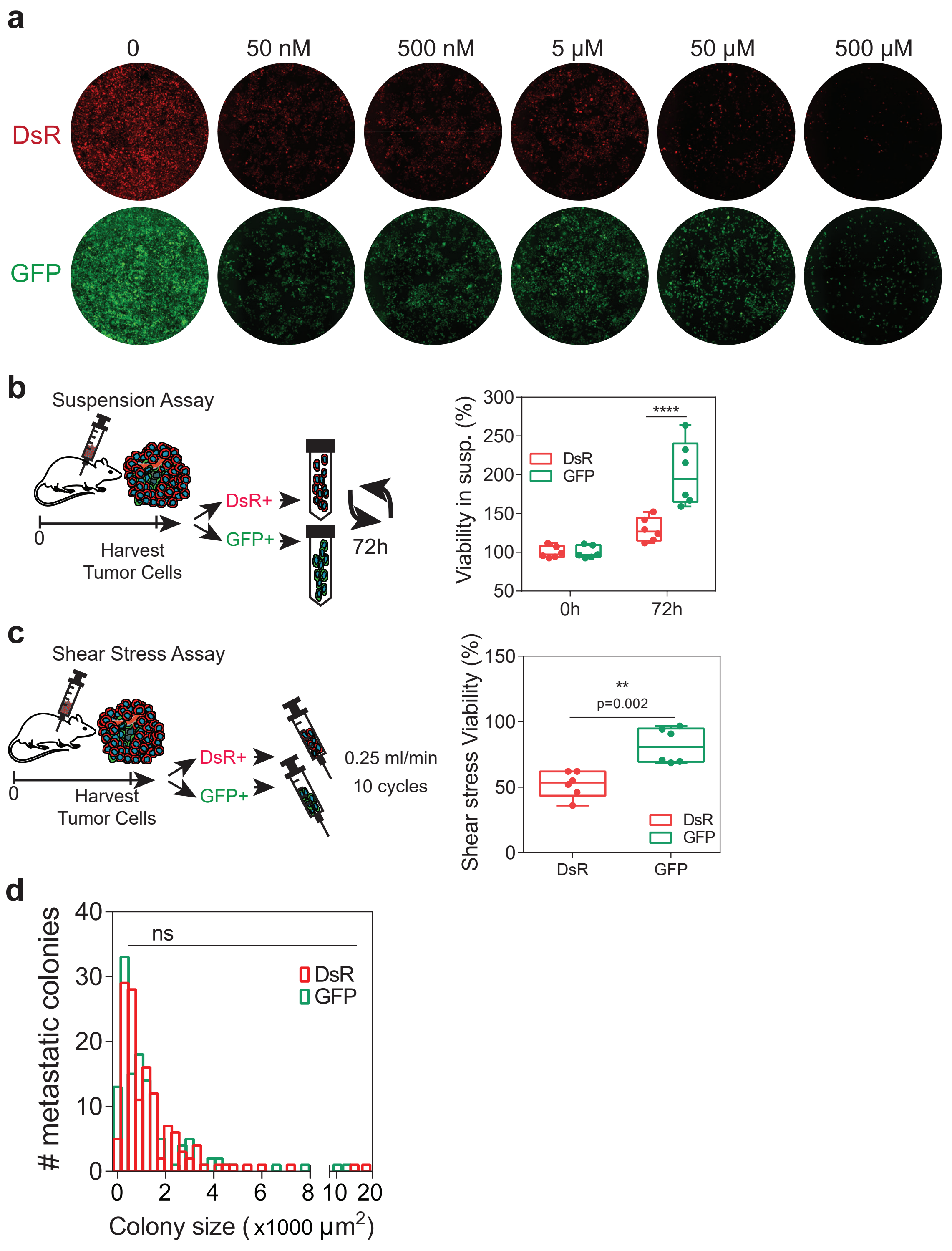

Supplementary Figure 11. Post-hypoxic cells have ROS-resistant phenotype. a, Fluorescent images of tumor-derived cells DsRed+ (DsR) or GFP+ imaged 48 h after 1 h of H_2O_2 treatment. **b,** Tumor-derived cells were cultured in centrifuge tubes that were maintained in constant rotation under normal tissue culture conditions for 72 h. After 72 h, cells were plated, and confluency was assessed the following day by image analysis (N=2, n=6); **** P<0.0001 GFP versus DsRed (Two-way ANOVA with Bonferroni post-test). The box extends from the 25th to 75th percentiles, the median is marked by the vertical line inside the box, and the whiskers represent the minimum and maximum points. **c,** Tumor-derived cells were trypsinized and resuspended in fresh media. Shear stress was simulated by passing the cell suspension ($1x10^5$/ml) at a constant flow rate of 0.25 ml/s using a syringe. After 10 cycles, cells were counted with Trypan Blue (N=1, n=6); **** P<0.0001 GFP versus DsRed (two-tailed t-test). Box and whiskers plot is defined as described in (**b**). **d,** Colony size distribution in lungs of mice 25 days after tail vein injection of DsRed+ and GFP+ tumor-derived cells (N=1, n=9); **** P<0.0001 GFP versus DsRed (Mann-Whitney test).

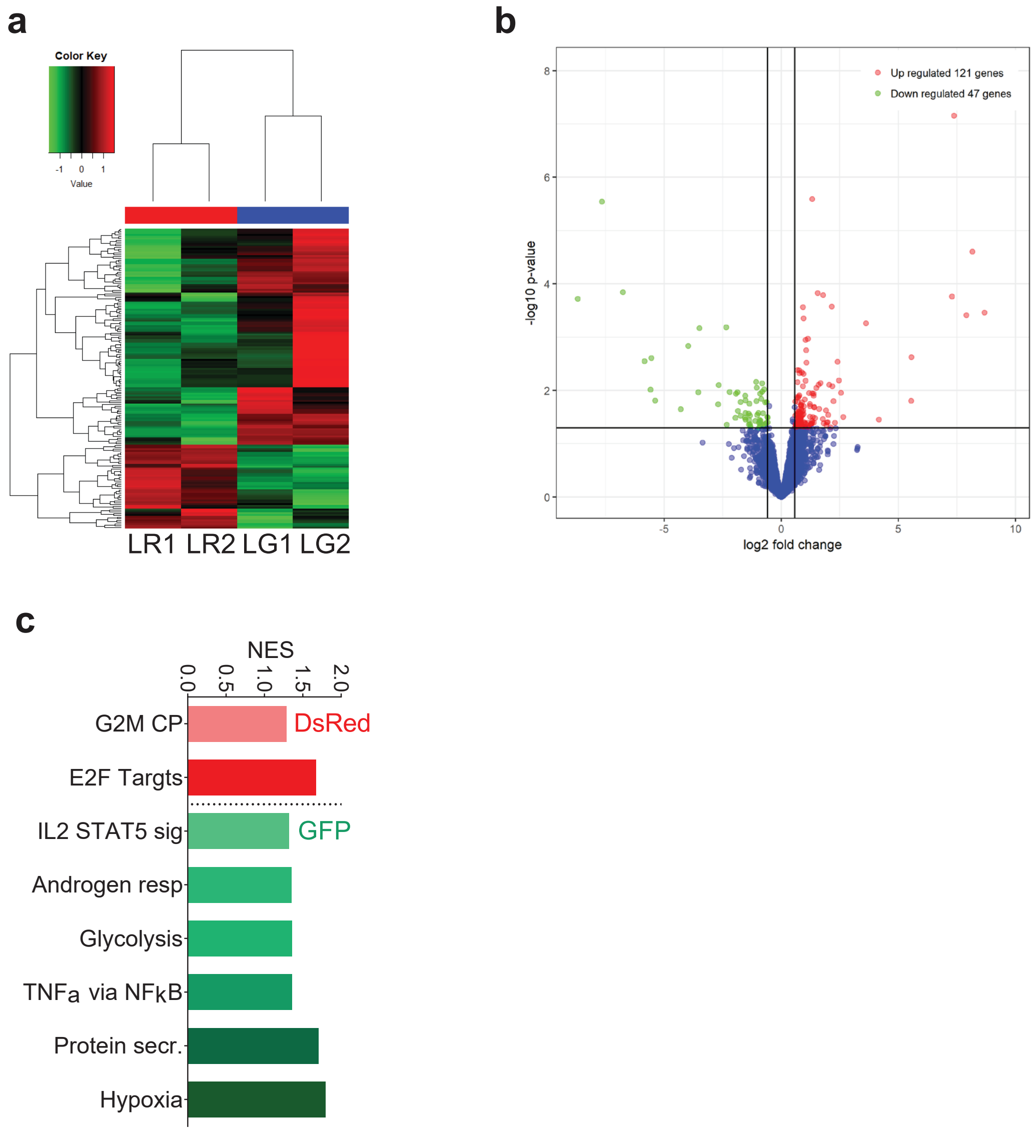

Supplementary Figure 12. RNA sequencing of metastatic cells in the lung. a, Heatmap of gene expression from RNA sequencing analysis which compares RNA expression in GFP+ metastatic cells (LG1 and LG2) to RNA expression in DsRed+ metastatic cells (LR1 and LR2). **b,** Volcano plot of gene expression from RNA sequencing analysis comparing GFP+ versus DsRed+ tumor cells. Green – down-regulated genes; Red – up-regulated genes (-1.5≥FC≥1.5) and p-value<0.05. **c,** Normalized enrichment scores (nominal p-value ≤ 0.05) derived from (Gene Set Enrichment Analysis) GSEA against Hallmark gene sets of DsRed+ and GFP+ cancer cells purified from resected lungs.

Supplementary Tables

Supplementary Table 1 – Primers used for qPCR.

Gene	FW	RV
Actin (Ms)	GGCTGTATTCCCCTCCATCG	CCAGTTGGTAACAATGCCATGT
CA9	GGATCTACCTACTGTTGAGGCT	CATAGCGCCAATGACTCTGGT
CRE	AGCCGAAATTGCCAGGAT	AACCAGCGTTTTCGTTCT
CP	CACGGGCAAGCACTGACTAA	GGGCCACCATATAAGCATCAAAC
DNAH11	CAACAGCTTACCTTTCACCTGA	TTCTTCCCATAAAGTAGCTTGCC
ENGLN3	GGCTGGGCAAATACTACGTCAA	CCTGTTCCATTTCCCGGATAG
GFP	GACGACGGCAACTACAAGAC	TCCTTGAAGTCGATGCCCTT
ITGA10	ACTTAGGTGACTACCAACGG	CCACAAGCACGAGACCAG
LOX	GTTCCAAGCTGGCTACTC	GGGTTGTCGTCAGAGTAC
P4HA1 (Ms)	AGCCACCATTTCAAACCCAGT	GCCAAGCACTTTTGCTAATTCTG

Supplementary Table 2 – Primers used for genotyping.

Sequence of interest	FW	RV
MMTV-PYMT	GGAAGCAAGTACTTCACAAGGG	GGAAAGTCACTAGGAGCAGGG
4xHRE-MinTK-CRE-ODD	CTGCTAACCATGTTCATGCCTTC	GACGATGAAGCATGTTTAGCTGGC

Supplementary Table 3 – Primers used for TLA sequencing.

Sequence of interest	FW	RV
Set 1 - bGlobin intron	AATACTCTGAGTCCAAACCG	TCGTTACAAATGCAAGCTAAA
Set 2 - Cre	GGAGTTTCAATACCGGAGAT	ATTACGTATATCCTGGCAGC

Supplementary Table 4 – Results of RNA sequencing analysis of tumor samples, presented as the fold change (FC) of tumor GFP+ (TG) cells over tumor DsRed+ (TR) cells. $-1.5 \geq FC \geq 1.5$ and p-value≤ 0.05.

Gene_symbol	NAME	Foldchange	PValue
PRH1	proline-rich protein HaeIII subfamily 1	-3.175	0.019
DGCR6	DiGeorge syndrome critical region gene 6	-2.939	0.028
HOXC10	homeobox C10	-2.742	0.020
S100A5	S100 calcium binding protein A5	-2.524	0.018
COMMD3-BMI1	COMMD3-BMI1 readthrough	-2.501	0.018

FAM45B	family with sequence similarity 45, member B (pseudogene)	-2.236	0.028
GRM5-AS1	GRM5 antisense RNA 1	-2.187	0.046
KRTAP2-3	keratin associated protein 2-3	-2.164	0.020
APITD1-CORT	APITD1-CORT readthrough	-2.133	0.039
RHEBL1	Ras homolog enriched in brain like 1	-2.041	0.039
B3GALT5	UDP-Gal:betaGlcNAc beta 1,3-galactosyltransferase, polypeptide 5	-2.032	0.047
RTEL1-TNFRSF6B	RTEL1-TNFRSF6B readthrough (NMD candidate)	-2.024	0.011
SPC24	SPC24, NDC80 kinetochore complex component	-1.896	0.023
FANCB	Fanconi anemia, complementation group B	-1.876	0.040
BATF3	basic leucine zipper transcription factor, ATF-like 3	-1.854	0.017
DYX1C1	dyslexia susceptibility 1 candidate 1	-1.851	0.032
LOC100507002	NA	-1.806	0.037
CBR3	carbonyl reductase 3	-1.737	0.018
FBP1	fructose-1,6-bisphosphatase 1	-1.733	0.037
C16orf59	chromosome 16 open reading frame 59	-1.724	0.017
C7orf55-LUC7L2	C7orf55-LUC7L2 readthrough	-1.721	0.007
MYBL2	v-myb avian myeloblastosis viral oncogene homolog-like 2	-1.714	0.005
MNS1	meiosis-specific nuclear structural 1	-1.685	0.046
MYZAP	myocardial zonula adherens protein	-1.676	0.042
PTPRR	protein tyrosine phosphatase, receptor type, R	-1.672	0.042
UBE2T	ubiquitin-conjugating enzyme E2T	-1.656	0.008
MND1	meiotic nuclear divisions 1	-1.650	0.018
HAS3	hyaluronan synthase 3	-1.635	0.037
CAMK4	calcium/calmodulin-dependent protein kinase IV	-1.608	0.045
GINS1	GINS complex subunit 1 (Psf1 homolog)	-1.597	0.020
CENPH	centromere protein H	-1.576	0.030
BIRC5	baculoviral IAP repeat containing 5	-1.569	0.020
TK1	thymidine kinase 1, soluble	-1.555	0.022
ATP6V0E2	ATPase, H+ transporting V0 subunit e2	-1.555	0.039
TIPIN	TIMELESS interacting protein	-1.548	0.033
ASF1B	anti-silencing function 1B histone chaperone	-1.546	0.034
GCSH	glycine cleavage system protein H (aminomethyl carrier)	-1.545	0.035
GINS2	GINS complex subunit 2 (Psf2 homolog)	-1.544	0.036
CDC45	cell division cycle 45	-1.540	0.027
ABRACL	ABRA C-terminal like	-1.539	0.031
CDC20	cell division cycle 20	-1.529	0.034
CDKN3	cyclin-dependent kinase inhibitor 3	-1.526	0.045
NRGN	neurogranin (protein kinase C substrate, RC3)	-1.522	0.028
PLK1	polo-like kinase 1	-1.517	0.024

CDCA3	cell division cycle associated 3	-1.514	0.043
ZWINT	ZW10 interacting kinetochore protein	-1.513	0.019
GTSE1	G-2 and S-phase expressed 1	-1.504	0.029
AURKB	aurora kinase B	-1.501	0.044
KIF24	kinesin family member 24	-1.500	0.046
ZEB2	zinc finger E-box binding homeobox 2	1.506	0.045
CCL5	chemokine (C-C motif) ligand 5	1.507	0.034
PARP14	poly (ADP-ribose) polymerase family, member 14	1.511	0.037
GABPB1-AS1	GABPB1 antisense RNA 1	1.511	0.029
SNED1	sushi, nidogen and EGF-like domains 1	1.516	0.028
LOC100289019	NA	1.517	0.046
BMI1	BMI1 proto-oncogene, polycomb ring finger	1.521	0.049
HSF4	heat shock transcription factor 4	1.525	0.041
TNXB	tenascin XB	1.528	0.023
IL1RAP	interleukin 1 receptor accessory protein	1.536	0.040
C1R	complement component 1, r subcomponent	1.548	0.042
LINC00649	long intergenic non-protein coding RNA 649	1.560	0.048
LRP1	low density lipoprotein receptor-related protein 1	1.562	0.018
MUC1	mucin 1, cell surface associated	1.564	0.015
FAM126B	family with sequence similarity 126, member B	1.570	0.048
ITGA10	integrin, alpha 10	1.572	0.016
SLFN5	schlafen family member 5	1.575	0.024
CFB	complement factor B	1.579	0.003
SLC26A1	solute carrier family 26 (anion exchanger), member 1	1.586	0.050
NDRG1	N-myc downstream regulated 1	1.586	0.008
SMG7	SMG7 nonsense mediated mRNA decay factor	1.589	0.007
PLSCR4	phospholipid scramblase 4	1.592	0.044
ZNF432	zinc finger protein 432	1.596	0.040
ZNF550	zinc finger protein 550	1.597	0.029
C6orf141	chromosome 6 open reading frame 141	1.598	0.018
HMBOX1	homeobox containing 1	1.602	0.044
ZNF791	zinc finger protein 791	1.608	0.041
ZNF426	zinc finger protein 426	1.612	0.045
PPARA	peroxisome proliferator-activated receptor alpha	1.619	0.030
KCNQ1OT1	KCNQ1 opposite strand/antisense transcript 1 (non-protein coding)	1.620	0.041
TMEM45A	transmembrane protein 45A	1.622	0.041
RNASE4	ribonuclease, RNase A family, 4	1.628	0.043
BMS1P4	BMS1 ribosome biogenesis factor pseudogene 4	1.633	0.040
IFI44L	interferon-induced protein 44-like	1.639	0.032
NEAT1	nuclear paraspeckle assembly transcript 1 (non-protein coding)	1.643	0.048

PCDHB14	protocadherin beta 14	1.649	0.030
LOC101928936	NA	1.654	0.036
EXD1	exonuclease 3'-5' domain containing 1	1.654	0.028
LRRC24	leucine rich repeat containing 24	1.656	0.033
LOC100131257	NA	1.657	0.033
RPL32P3	ribosomal protein L32 pseudogene 3	1.658	0.048
TTC30A	tetratricopeptide repeat domain 30A	1.662	0.035
ZNF81	zinc finger protein 81	1.666	0.039
ARPC4-TTLL3	ARPC4-TTLL3 readthrough	1.666	0.048
UCKL1-AS1	UCKL1 antisense RNA 1	1.671	0.015
LOC90834	NA	1.678	0.027
MALAT1	metastasis associated lung adenocarcinoma transcript 1 (non-protein coding)	1.681	0.021
ASTN2	astrotactin 2	1.692	0.031
LOC100630923	NA	1.693	0.026
LOC100288069	NA	1.695	0.034
FN1	fibronectin 1	1.695	0.002
PLA2R1	phospholipase A2 receptor 1, 180kDa	1.698	0.010
BMS1P5	BMS1 ribosome biogenesis factor pseudogene 5	1.702	0.018
BMS1P6	BMS1 ribosome biogenesis factor pseudogene 6	1.705	0.018
CA9	carbonic anhydrase IX	1.708	0.006
ZNF91	zinc finger protein 91	1.715	0.019
LOC646214	NA	1.720	0.042
LOC101928600	NA	1.726	0.017
FAM13A	family with sequence similarity 13, member A	1.728	0.012
HSD3BP4	hydroxy-delta-5-steroid dehydrogenase, 3 beta, pseudogene 4	1.730	0.031
EXPH5	exophilin 5	1.736	0.010
RBM43	RNA binding motif protein 43	1.739	0.043
LOC646471	NA	1.739	0.049
FAM63B	family with sequence similarity 63, member B	1.741	0.049
ZNF708	zinc finger protein 708	1.743	0.029
CCDC68	coiled-coil domain containing 68	1.743	0.048
IL18BP	interleukin 18 binding protein	1.755	0.018
LINC00506	long intergenic non-protein coding RNA 506	1.755	0.012
CPEB4	cytoplasmic polyadenylation element binding protein 4	1.759	0.015
PTGFR	prostaglandin F receptor (FP)	1.762	0.048
LINC00174	long intergenic non-protein coding RNA 174	1.764	0.042
LOC101926889	NA	1.764	0.020
CAPRIN2	caprin family member 2	1.766	0.016
PTGS2	prostaglandin-endoperoxide synthase 2 (prostaglandin G/H synthase and cyclooxygenase)	1.774	0.001
USP32P2	ubiquitin specific peptidase 32 pseudogene 2	1.775	0.049

ANKRD36B	ankyrin repeat domain 36B	1.788	0.012
DNAH1	dynein, axonemal, heavy chain 1	1.790	0.006
PPFIA4	protein tyrosine phosphatase, receptor type, f polypeptide (PTPRF), interacting protein (liprin), alpha 4	1.792	0.009
KDM7A	lysine (K)-specific demethylase 7A	1.799	0.031
ZNF224	zinc finger protein 224	1.802	0.030
ZNF460	zinc finger protein 460	1.811	0.014
ZNF235	zinc finger protein 235	1.813	0.034
TSSK3	testis-specific serine kinase 3	1.814	0.030
ZCWPW1	zinc finger, CW type with PWWP domain 1	1.816	0.049
LOC101928137	NA	1.832	0.041
ZNF121	zinc finger protein 121	1.833	0.017
AMT	aminomethyltransferase	1.841	0.022
ZNF439	zinc finger protein 439	1.842	0.048
KBTBD12	kelch repeat and BTB (POZ) domain containing 12	1.843	0.009
GRTP1	growth hormone regulated TBC protein 1	1.846	0.021
MTUS1	microtubule associated tumor suppressor 1	1.847	0.017
DUXA	double homeobox A	1.849	0.042
TRIM66	tripartite motif containing 66	1.857	0.006
OTUD6A	OTU deubiquitinase 6A	1.876	0.026
IDI2	isopentenyl-diphosphate delta isomerase 2	1.880	0.031
MMP10	matrix metallopeptidase 10	1.887	0.047
EGLN3	egl-9 family hypoxia-inducible factor 3	1.911	0.010
SCG2	secretogranin II	1.915	0.038
LOC401320	NA	1.917	0.019
DNAH11	dynein, axonemal, heavy chain 11	1.920	0.014
MAP1LC3C	microtubule-associated protein 1 light chain 3 gamma	1.924	0.005
ZNF204P	zinc finger protein 204, pseudogene	1.932	0.034
LRRC37A	leucine rich repeat containing 37A	1.934	0.050
PARGP1	poly (ADP-ribose) glycohydrolase pseudogene 1	1.942	0.032
LINC00319	long intergenic non-protein coding RNA 319	1.945	0.048
GOLGA8B	golgin A8 family, member B	1.949	0.015
SEC31B	SEC31 homolog B, COPII coat complex component	1.954	0.004
AHRR	aryl-hydrocarbon receptor repressor	1.956	0.025
KIAA1614	KIAA1614	1.965	0.021
INHA	inhibin, alpha	1.969	0.033
NYAP2	neuronal tyrosine-phosphorylated phosphoinositide-3-kinase adaptor 2	1.969	0.037
WNT2B	wingless-type MMTV integration site family, member 2B	1.989	0.026
CCDC144B	coiled-coil domain containing 144B (pseudogene)	1.999	0.016

PTGS1	prostaglandin-endoperoxide synthase 1 (prostaglandin G/H synthase and cyclooxygenase)	2.007	0.034
C9orf9	chromosome 9 open reading frame 9	2.008	0.029
FAM227A	family with sequence similarity 227, member A	2.010	0.030
SH3D21	SH3 domain containing 21	2.016	0.022
LOC100132062	NA	2.017	0.001
TMEM145	transmembrane protein 145	2.032	0.028
SSC5D	scavenger receptor cysteine rich family, 5 domains	2.052	0.005
TNFSF10	tumor necrosis factor (ligand) superfamily, member 10	2.055	0.028
LOC100190986	NA	2.057	0.008
TTN	titin	2.081	0.012
LOC100131564	NA	2.084	0.023
LOX	lysyl oxidase	2.105	0.002
NLRP12	NLR family, pyrin domain containing 12	2.114	0.002
C1S	complement component 1, s subcomponent	2.117	0.003
LINC00458	long intergenic non-protein coding RNA 458	2.117	0.008
RPLP0P2	ribosomal protein, large, P0 pseudogene 2	2.122	0.022
DNAH7	dynein, axonemal, heavy chain 7	2.131	0.007
ZNF616	zinc finger protein 616	2.150	0.018
CSAD	cysteine sulfinic acid decarboxylase	2.173	0.003
USP32P1	ubiquitin specific peptidase 32 pseudogene 1	2.187	0.008
GUSBP3	glucuronidase, beta pseudogene 3	2.190	0.002
FAM71F2	family with sequence similarity 71, member F2	2.194	0.025
PCDHGB3	protocadherin gamma subfamily B, 3	2.202	0.036
ZC3H12B	zinc finger CCCH-type containing 12B	2.204	0.034
RORA	RAR-related orphan receptor A	2.213	0.015
ATP6V1G2-DDX39B	ATP6V1G2-DDX39B readthrough (NMD candidate)	2.226	0.002
SMG1P1	SMG1 pseudogene 1	2.229	0.001
JAK3	Janus kinase 3	2.233	0.008
ABCA1	ATP-binding cassette, sub-family A (ABC1), member 1	2.235	0.009
CNTN4	contactin 4	2.236	0.022
ZNF818P	zinc finger protein 818, pseudogene	2.257	0.014
LRRC56	leucine rich repeat containing 56	2.258	0.013
LOC100506551	NA	2.260	0.041
SLC9A4	solute carrier family 9, subfamily A (NHE4, cation proton antiporter 4), member 4	2.263	0.015
BACE1-AS	BACE1 antisense RNA	2.265	0.035
LINC00926	long intergenic non-protein coding RNA 926	2.267	0.026
KCND1	potassium channel, voltage gated Shal related subfamily D, member 1	2.270	0.013
LOC100996251	NA	2.270	0.038
SPERT	spermatid associated	2.279	0.032
TBX6	T-box 6	2.288	0.011

MST1	macrophage stimulating 1	2.325	0.001
RASA4B	RAS p21 protein activator 4B	2.331	0.019
COL11A2	collagen, type XI, alpha 2	2.363	0.043
LRRC3	leucine rich repeat containing 3	2.402	0.027
HS3ST3A1	heparan sulfate (glucosamine) 3-O-sulfotransferase 3A1	2.404	0.049
PKHD1	polycystic kidney and hepatic disease 1 (autosomal recessive)	2.425	0.032
GSDMB	gasdermin B	2.428	0.041
LINC01207	long intergenic non-protein coding RNA 1207	2.440	0.032
ELOVL7	ELOVL fatty acid elongase 7	2.450	0.016
HIST2H3D	histone cluster 2, H3d	2.457	0.006
TMEM178A	transmembrane protein 178A	2.497	0.029
FRY	furry homolog (Drosophila)	2.500	0.003
MSS51	MSS51 mitochondrial translational activator	2.534	0.016
LOC100130451	NA	2.551	0.016
MSH5-SAPCD1	MSH5-SAPCD1 readthrough (NMD candidate)	2.564	0.021
SKIDA1	SKI/DACH domain containing 1	2.565	0.036
MUC15	mucin 15, cell surface associated	2.577	0.020
EBLN2	endogenous Bornavirus-like nucleoprotein 2	2.623	0.046
SEMA4A	sema domain, immunoglobulin domain (Ig), transmembrane domain (TM) and short cytoplasmic domain, (semaphorin) 4A	2.669	0.013
SENP3-EIF4A1	SENP3-EIF4A1 readthrough (NMD candidate)	2.737	0.002
GPC2	glypican 2	2.760	0.029
TMEM236	transmembrane protein 236	2.770	0.020
KCNQ3	potassium channel, voltage gated KQT-like subfamily Q, member 3	2.795	0.002
DNAJC9-AS1	DNAJC9 antisense RNA 1	2.803	0.017
APOL4	apolipoprotein L, 4	2.804	0.002
ANKRD20A12P	ankyrin repeat domain 20 family, member A12, pseudogene	2.824	0.014
LOC100289511	NA	2.837	0.002
PCDHGA7	protocadherin gamma subfamily A, 7	2.886	0.009
LOC101929698	NA	2.892	0.011
BAGE2	B melanoma antigen family, member 2	2.896	0.014
BAGE3	B melanoma antigen family, member 3	2.896	0.014
LOC100505817	NA	2.913	0.025
AGBL2	ATP/GTP binding protein-like 2	2.926	0.026
CAND2	cullin-associated and neddylation-dissociated 2 (putative)	2.963	0.009
MIRLET7DHG	MIRLET7D host gene	3.008	0.015
CCDC183	coiled-coil domain containing 183	3.034	0.002
GADD45G	growth arrest and DNA-damage-inducible, gamma	3.137	0.015
LOC100996291	NA	3.166	0.039

SLC7A11-AS1	SLC7A11 antisense RNA 1	3.207	0.002
ANKRD20A5P	ankyrin repeat domain 20 family, member A5, pseudogene	3.228	0.001
CCDC144CP	coiled-coil domain containing 144C, pseudogene	3.274	0.020
ADAM28	ADAM metallopeptidase domain 28	3.286	0.026
FER1L4	fer-1-like family member 4, pseudogene (functional)	3.308	0.000
BREA2	breast cancer estrogen-induced apoptosis 2	3.309	0.008
CDKN2B-AS1	CDKN2B antisense RNA 1	3.742	0.034
PSORS1C2	psoriasis susceptibility 1 candidate 2	3.771	0.014
GCOM1	GRINL1A complex locus 1	4.241	0.039
RAPGEF4	Rap guanine nucleotide exchange factor (GEF) 4	4.260	0.003
LINC01209	long intergenic non-protein coding RNA 1209	4.448	0.008
CHRNA10	cholinergic receptor, nicotinic, alpha 10 (neuronal)	4.666	0.004
RBAK-RBAKDN	RBAK-RBAKDN readthrough	4.789	0.029
LOC100272216	NA	4.898	0.004
CTAGE8	CTAGE family, member 8	5.148	0.001
PCDHGB6	protocadherin gamma subfamily B, 6	5.582	0.000
CP	ceruloplasmin (ferroxidase)	5.914	0.000
JMJD7-PLA2G4B	JMJD7-PLA2G4B readthrough	6.440	0.006
CAMK2B	calcium/calmodulin-dependent protein kinase II beta	6.537	0.001
STAC2	SH3 and cysteine rich domain 2	10.771	0.000
C8orf44-SGK3	C8orf44-SGK3 readthrough	11.959	0.000
SPECC1L-ADORA2A	SPECC1L-ADORA2A readthrough (NMD candidate)	131.588	0.000
NARR	NA	149.577	0.000

Supplementary Table 5 – GSEA Enrichment Analysis investigating Hallmark Pathways in sorted tumor samples GFP+ (TG) or DsRed+ (TR). Highlighted in green are the hallmarks with p-value≤0.05 and FDR≤0.25.

a) Enrichment in GFP+ tumor cells (TG)

NAME	SIZE	ES	NES	NOM p-val	FDR q-val	FWER p-val	RANK AT MAX
HALLMARK_HYPOXIA	199	0.461273	1.530811	0.001221	0.104524	0.13	5992
HALLMARK_KRAS_SIGNALING_UP	199	0.387559	1.283896	0.035758	0.875634	0.908	5451
HALLMARK_UV_RESPONSE_DN	144	0.392945	1.270089	0.064677	0.663513	0.939	6165
HALLMARK_INTERFERON_GAMMA_RESPONSE	197	0.363632	1.208407	0.098914	0.860122	0.993	5079
HALLMARK_TGF_BETA_SIGNALING	54	0.421787	1.189926	0.2	0.802027	0.999	6023

HALLMARK_COAGULATION	136	0.356738	1.148072	0.199208	0.938739	1	5288
HALLMARK_KRAS_SIGNALING_DN	195	0.342328	1.129536	0.190876	0.917909	1	4585
HALLMARK_MYOGENESIS	200	0.326941	1.09413	0.259901	1	1	4553
HALLMARK_APICAL_SURFACE	44	0.396621	1.091559	0.323708	0.922099	1	4835
HALLMARK_TNFA_SIGNALING_VIA_NFKB	199	0.327551	1.090394	0.265133	0.834639	1	6033
HALLMARK_HEDGEHOG_SIGNALING	36	0.407156	1.076439	0.358255	0.830197	1	4046
HALLMARK_EPITHELIAL_MESENCHYMAL_TRANSITION	198	0.322281	1.073618	0.308733	0.773852	1	5955
HALLMARK_INTERFERON_ALPHA_RESPONSE	94	0.348565	1.058874	0.343124	0.780835	1	5910
HALLMARK_ANGIOGENESIS	36	0.399743	1.052746	0.381847	0.751164	1	5681
HALLMARK_PROTEIN_SECRETION	96	0.34314	1.050399	0.357992	0.711255	1	5081
HALLMARK_INFLAMMATORY_RESPONSE	198	0.277283	0.917792	0.665868	1	1	4257
HALLMARK_ANDROGEN_RESPONSE	101	0.293542	0.901939	0.666234	1	1	4079
HALLMARK_IL6_JAK_STAT3_SIGNALING	87	0.294815	0.892237	0.673228	1	1	4320
HALLMARK_COMPLEMENT	196	0.259563	0.858963	0.812424	1	1	4294
HALLMARK_HEME_METABOLISM	195	0.254664	0.843726	0.845411	1	1	5951
HALLMARK_IL2_STAT5_SIGNALING	199	0.247446	0.827813	0.881628	1	1	5033
HALLMARK_APOPTOSIS	160	0.253262	0.825132	0.862944	1	1	4654
HALLMARK_MITOTIC_SPINDLE	198	0.241574	0.805556	0.923821	1	1	6005
HALLMARK_BILE_ACID_METABOLISM	112	0.256948	0.80065	0.891821	1	1	3647
HALLMARK_ALLOGRAFT_REJECTION	200	0.232496	0.775823	0.967312	1	1	5182
HALLMARK_GLYCOLYSIS	199	0.224532	0.75346	0.979592	1	1	4570
HALLMARK_APICAL_JUNCTION	200	0.224096	0.748908	0.98652	1	1	4442
HALLMARK_NOTCH_SIGNALING	32	0.287019	0.737307	0.8784	1	1	7614
HALLMARK_ESTROGEN_RESPONSE_EARLY	200	0.218621	0.720617	0.991667	1	1	5797
HALLMARK_PANCREAS_BETA_CELLS	40	0.248845	0.670079	0.968404	0.993925	1	2624

b) Enrichment in DsRed+ tumor cells (TR)

NAME	SIZE	ES	NES	NOM p-val	FDR q-val	FWER p-val	RANK AT MAX
HALLMARK_E2F_TARGETS	199	0.715396	2.744474	0	0	0	4045
HALLMARK_MYC_TARGETS_V1	199	0.71203	2.711706	0	0	0	5035
HALLMARK_OXIDATIVE_PHOSPHORYLATION	199	0.680298	2.605333	0	0	0	5738
HALLMARK_G2M_CHECKPOINT	200	0.61316	2.363222	0	0	0	4086
HALLMARK_MYC_TARGETS_V2	58	0.70224	2.275379	0	0	0	5675
HALLMARK_DNA_REPAIR	143	0.587405	2.171609	0	0	0	5461
HALLMARK_REACTIVE_OXIGEN_SPECIES_PATHWAY	47	0.585989	1.792009	0	2.60E-04	0.001	5720
HALLMARK_MTORC1_SIGNALING	199	0.447233	1.735374	0	0.001346	0.006	6592
HALLMARK_FATTY_ACID_METABOLISM	158	0.436888	1.639641	0	0.003254	0.017	5239
HALLMARK_ADIPOGENESIS	196	0.419265	1.608216	0	0.004531	0.027	6430
HALLMARK_UNFOLDED_PROTEIN_RESPONSE	112	0.422675	1.534899	0	0.007613	0.047	6435
HALLMARK_ESTROGEN_RESPONSE_LATE	199	0.393403	1.489699	0	0.009556	0.064	5377
HALLMARK_XENOBIOTIC_METABOLISM	199	0.37087	1.428545	0	0.015292	0.109	5004
HALLMARK_PEROXISOME	103	0.388516	1.348908	0.011494	0.035281	0.25	5884
HALLMARK_UV_RESPONSE_UP	156	0.345333	1.289185	0.037433	0.056717	0.385	5420
HALLMARK_PI3K_AKT_MTOR_SIGNALING	105	0.348908	1.230824	0.087591	0.089077	0.553	4536
HALLMARK_SPERMATOGENESIS	133	0.331812	1.226506	0.082192	0.086546	0.567	2508
HALLMARK_CHOLESTEROL_HOMEOSTASIS	73	0.355196	1.173443	0.140893	0.130512	0.728	4675
HALLMARK_P53_PATHWAY	199	0.285176	1.08542	0.21134	0.245589	0.93	5709
HALLMARK_WNT_BETA_CATENIN_SIGNALING	42	0.223649	0.656045	0.98806	0.996677	1	4673

Supplemental Table 6 – Results of VennPlex analysis overlapping gene expression (-1.5≥FC≥1.5, p-value≤0.05) in primary tumor (TG vs TR) with hypoxic induction in culture conditions (1% vs 20% O_2).

TG/TR		TG/TR ∩ 1%/20%		
Up-regulated Total = 164	*Down-regulated Total = 43*	***Up-regulated Total = 41***	*Contra-regulated Total = 12*	***Down-regulated Total = 3***

ZEB2	PRH1	**LRP1**	B3GALT5	**COMMD3-BMI1**
CCL5	DGCR6	**NDRG1**	RTEL1-TNFRSF6B	**FAM45B**
PARP14	HOXC10	**TMEM45A**	DYX1C1	**CENPH**
GABPB1-AS1	S100A5	**LRRC24**	SNED1	
BMI1	GRM5-AS1	**ARPC4-TTLL3**	LOC100289019	
HSF4	KRTAP2-3	**CA9**	LOC100630923	
TNXB	APITD1-CORT	**HSD3BP4**	IL18BP	
IL1RAP	RHEBL1	**RBM43**	SCG2	
C1R	SPC24	**LOC646471**	KIAA1614	
LINC00649	FANCB	**PTGFR**	PCDHGB3	
MUC1	BATF3	**LINC00174**	SENP3-EIF4A1	
FAM126B	LOC100507002	**USP32P2**	CHRNA10	
ITGA10	CBR3	**PPFIA4**		
SLFN5	FBP1	**KDM7A**		
CFB	C16orf59	**LOC101928137**		
SLC26A1	C7orf55-LUC7L2	**ZNF439**		
SMG7	MYBL2	**OTUD6A**		
PLSCR4	MNS1	**EGLN3**		
ZNF432	MYZAP	**DNAH11**		
ZNF550	PTPRR	**PARGP1**		
C6orf141	UBE2T	**C9orf9**		
HMBOX1	MND1	**LOX**		
ZNF791	HAS3	**RORA**		
ZNF426	CAMK4	**SMG1P1**		
PPARA	GINS1	**JAK3**		
KCNQ1OT1	BIRC5	**ZNF818P**		
RNASE4	TK1	**SLC9A4**		
BMS1P4	ATP6V0E2	**LINC00926**		
IFI44L	TIPIN	**SPERT**		
NEAT1	ASF1B	**LRRC3**		
PCDHB14	GCSH	**MUC15**		
LOC101928936	GINS2	**KCNQ3**		
EXD1	CDC45	**DNAJC9-AS1**		
LOC100131257	ABRACL	**PCDHGA7**		
RPL32P3	CDC20	**ANKRD20A5P**		
TTC30A	CDKN3	**FER1L4**		
ZNF81	NRGN	**RAPGEF4**		
UCKL1-AS1	PLK1	**LOC100272216**		
LOC90834	CDCA3	**PCDHGB6**		
MALAT1	ZWINT	**JMJD7-PLA2G4B**		

ASTN2	GTSE1	**SPECC1L-ADORA2A**		
LOC100288069	AURKB			
FN1	KIF24			
PLA2R1				
BMS1P5				
BMS1P6				
ZNF91				
LOC646214				
LOC101928600				
FAM13A				
EXPH5				
FAM63B				
ZNF708				
CCDC68				
LINC00506				
CPEB4				
LOC101926889				
CAPRIN2				
PTGS2				
ANKRD36B				
DNAH1				
ZNF224				
ZNF460				
ZNF235				
TSSK3				
ZCWPW1				
ZNF121				
AMT				
KBTBD12				
GRTP1				
MTUS1				
DUXA				
TRIM66				
IDI2				
MMP10				
LOC401320				
MAP1LC3C				
ZNF204P				
LRRC37A				
LINC00319				
GOLGA8B				

SEC31B				
AHRR				
INHA				
NYAP2				
WNT2B				
CCDC144B				
PTGS1				
FAM227A				
SH3D21				
LOC100132062				
TMEM145				
SSC5D				
TNFSF10				
LOC100190986				
TTN				
LOC100131564				
NLRP12				
C1S				
LINC00458				
RPLP0P2				
DNAH7				
ZNF616				
CSAD				
USP32P1				
GUSBP3				
FAM71F2				
ZC3H12B				
ATP6V1G2-DDX39B				
ABCA1				
CNTN4				
LRRC56				
LOC100506551				
BACE1-AS				
KCND1				
LOC100996251				
TBX6				
MST1				
RASA4B				
COL11A2				
HS3ST3A1				
PKHD1				

GSDMB				
LINC01207				
ELOVL7				
HIST2H3D				
TMEM178A				
FRY				
MSS51				
LOC100130451				
MSH5-SAPCD1				
SKIDA1				
EBLN2				
SEMA4A				
GPC2				
TMEM236				
APOL4				
ANKRD20A12P				
LOC100289511				
LOC101929698				
BAGE2				
BAGE3				
LOC100505817				
AGBL2				
CAND2				
MIRLET7DHG				
CCDC183				
GADD45G				
LOC100996291				
SLC7A11-AS1				
CCDC144CP				
ADAM28				
BREA2				
CDKN2B-AS1				
PSORS1C2				
GCOM1				
LINC01209				
RBAK-RBAKDN				
CTAGE8				
CP				
CAMK2B				
STAC2				
C8orf44-SGK3				

NARR				

Supplementary Table 7 – Results of RNA sequencing analysis of lung samples, presented as the fold change (FC) of metastatic GFP+ (LG) cells over DsRed+ (LR) cells. -1.5≥FC≥1.5 and p-value≤0.05.

Gene_symbol	NAME	FC	PValue
LOC642423	NA	-15.815	0.001
LDLRAD2	low density lipoprotein receptor class A domain containing 2	-11.331	0.001
HNRNPA1P10	heterogeneous nuclear ribonucleoprotein A1 pseudogene 10	-6.514	0.018
PLA2G4B	phospholipase A2, group IVB (cytosolic)	-5.103	0.001
LOC101928295	NA	-3.818	0.011
NCRUPAR	non-protein coding RNA, upstream of F2R/PAR1	-3.626	0.024
FLT4	fms-related tyrosine kinase 4	-3.345	0.030
SLC34A2	solute carrier family 34 (type II sodium/phosphate cotransporter), member 2	-3.336	0.016
CGB	chorionic gonadotropin, beta polypeptide	-2.945	0.033
FIRRE	firre intergenic repeating RNA element	-2.912	0.012
ALPPL2	alkaline phosphatase, placental-like 2	-2.894	0.035
GRM5-AS1	GRM5 antisense RNA 1	-2.885	0.026
ZNF852	zinc finger protein 852	-2.883	0.015
ANPEP	alanyl (membrane) aminopeptidase	-2.637	0.027
FRMPD3	FERM and PDZ domain containing 3	-2.616	0.043
SCN11A	sodium channel, voltage gated, type XI alpha subunit	-2.589	0.036
OLFML2B	olfactomedin-like 2B	-2.568	0.018
POM121L10P	POM121 transmembrane nucleoporin-like 10, pseudogene	-2.526	0.030
ZNF684	zinc finger protein 684	-2.526	0.043
GPC2	glypican 2	-2.512	0.048
HOMEZ	homeobox and leucine zipper encoding	-2.495	0.048
ZNF365	zinc finger protein 365	-2.160	0.037
PCDHGA3	protocadherin gamma subfamily A, 3	-2.115	0.007
FAM86JP	family with sequence similarity 86, member J, pseudogene	-2.097	0.016
FGD3	FYVE, RhoGEF and PH domain containing 3	-2.080	0.009
VIPR1	vasoactive intestinal peptide receptor 1	-2.040	0.029
LRRN4CL	LRRN4 C-terminal like	-1.975	0.026
KCNJ12	potassium channel, inwardly rectifying subfamily J, member 12	-1.942	0.049
RAB37	RAB37, member RAS oncogene family	-1.937	0.040
PCDHGA12	protocadherin gamma subfamily A, 12	-1.914	0.036
OXTR	oxytocin receptor	-1.887	0.039
PDGFRB	platelet-derived growth factor receptor, beta polypeptide	-1.866	0.012
RASL10A	RAS-like, family 10, member A	-1.866	0.046

APOBEC3G	apolipoprotein B mRNA editing enzyme, catalytic polypeptide-like 3G	-1.856	0.037
SSC5D	scavenger receptor cysteine rich family, 5 domains	-1.815	0.014
HCP5	HLA complex P5 (non-protein coding)	-1.770	0.011
NCF2	neutrophil cytosolic factor 2	-1.763	0.007
ARHGAP5-AS1	ARHGAP5 antisense RNA 1 (head to head)	-1.716	0.027
PAPLN	papilin, proteoglycan-like sulfated glycoprotein	-1.692	0.045
GDF15	growth differentiation factor 15	-1.665	0.010
HAS3	hyaluronan synthase 3	-1.648	0.043
PLCG2	phospholipase C, gamma 2 (phosphatidylinositol-specific)	-1.621	0.017
MYRF	myelin regulatory factor	-1.569	0.041
LAPTM5	lysosomal protein transmembrane 5	-1.544	0.017
SHROOM2	shroom family member 2	-1.510	0.031
ADAMTS15	ADAM metallopeptidase with thrombospondin type 1 motif, 15	-1.505	0.040
TNFAIP8L1	tumor necrosis factor, alpha-induced protein 8-like 1	-1.504	0.032
PKP2	plakophilin 2	1.506	0.029
ALDOC	aldolase C, fructose-bisphosphate	1.510	0.016
TPD52	tumor protein D52	1.518	0.049
KLF5	Kruppel-like factor 5 (intestinal)	1.520	0.046
RFK	riboflavin kinase	1.526	0.043
HEATR5A	HEAT repeat containing 5A	1.534	0.049
F8	coagulation factor VIII, procoagulant component	1.542	0.046
SOWAHC	sosondowah ankyrin repeat domain family member C	1.545	0.046
PSMD5-AS1	PSMD5 antisense RNA 1 (head to head)	1.555	0.050
DBNDD2	dysbindin (dystrobrevin binding protein 1) domain containing 2	1.573	0.013
SBF2-AS1	SBF2 antisense RNA 1	1.574	0.043
IGFBP1	insulin-like growth factor binding protein 1	1.580	0.028
AKAP12	A kinase (PRKA) anchor protein 12	1.583	0.033
BNIP3L	BCL2/adenovirus E1B 19kDa interacting protein 3-like	1.594	0.049
L2HGDH	L-2-hydroxyglutarate dehydrogenase	1.600	0.048
ZMYM1	zinc finger, MYM-type 1	1.603	0.048
CXCR4	chemokine (C-X-C motif) receptor 4	1.604	0.039
GTF2H2	general transcription factor IIH, polypeptide 2, 44kDa	1.609	0.041
BNIP3	BCL2/adenovirus E1B 19kDa interacting protein 3	1.610	0.004
ADM	adrenomedullin	1.615	0.007
DTWD1	DTW domain containing 1	1.618	0.045
SLC36A4	solute carrier family 36 (proton/amino acid symporter), member 4	1.626	0.027
MXI1	MAX interactor 1, dimerization protein	1.628	0.016
RNASE4	ribonuclease, RNase A family, 4	1.635	0.038
ZNF521	zinc finger protein 521	1.636	0.025
CRIP2	cysteine-rich protein 2	1.641	0.014

FAM13A	family with sequence similarity 13, member A	1.650	0.022
VLDLR	very low density lipoprotein receptor	1.651	0.032
ZXDA	zinc finger, X-linked, duplicated A	1.652	0.034
ZBTB21	zinc finger and BTB domain containing 21	1.657	0.035
TMEM181	transmembrane protein 181	1.669	0.044
ERO1L	NA	1.670	0.013
KIAA0232	KIAA0232	1.673	0.043
FAM45A	family with sequence similarity 45, member A	1.691	0.025
MIR210HG	MIR210 host gene	1.700	0.012
PDGFB	platelet-derived growth factor beta polypeptide	1.702	0.004
LRRC8B	leucine rich repeat containing 8 family, member B	1.703	0.048
C10orf10	chromosome 10 open reading frame 10	1.707	0.005
ARSK	arylsulfatase family, member K	1.710	0.030
TP53INP1	tumor protein p53 inducible nuclear protein 1	1.712	0.048
EML6	echinoderm microtubule associated protein like 6	1.724	0.046
ZNF766	zinc finger protein 766	1.739	0.034
MTX3	metaxin 3	1.739	0.026
SLC25A40	solute carrier family 25, member 40	1.751	0.045
WHAMMP1	WAS protein homolog associated with actin, golgi membranes and microtubules pseudogene 1	1.758	0.047
LIMCH1	LIM and calponin homology domains 1	1.758	0.036
SLC35A3	solute carrier family 35 (UDP-N-acetylglucosamine (UDP-GlcNAc) transporter), member A3	1.759	0.034
FKBP9P1	FK506 binding protein 9 pseudogene 1	1.769	0.032
POLK	polymerase (DNA directed) kappa	1.771	0.043
DNAH11	dynein, axonemal, heavy chain 11	1.789	0.033
COX10-AS1	COX10 antisense RNA 1	1.790	0.041
PDE1C	phosphodiesterase 1C, calmodulin-dependent 70kDa	1.790	0.046
TPPP	tubulin polymerization promoting protein	1.794	0.018
VGLL3	vestigial-like family member 3	1.795	0.019
SOCS4	suppressor of cytokine signaling 4	1.799	0.022
CCDC176	coiled-coil domain containing 176	1.804	0.019
RIMKLA	ribosomal modification protein rimK-like family member A	1.818	0.040
GOLGA6L9	golgin A6 family-like 9	1.823	0.030
HS3ST3B1	heparan sulfate (glucosamine) 3-O-sulfotransferase 3B1	1.840	0.017
IGFBP3	insulin-like growth factor binding protein 3	1.840	0.005
NPIPB6	nuclear pore complex interacting protein family, member B6	1.846	0.025
CSGALNACT1	chondroitin sulfate N-acetylgalactosaminyltransferase 1	1.854	0.047
IDI2	isopentenyl-diphosphate delta isomerase 2	1.859	0.029
BCL2A1	BCL2-related protein A1	1.879	0.046
TTN	titin	1.889	0.047
NDRG1	N-myc downstream regulated 1	1.894	0.000

SLIT2	slit guidance ligand 2	1.900	0.008
PRPF39	pre-mRNA processing factor 39	1.908	0.024
PTGS2	prostaglandin-endoperoxide synthase 2 (prostaglandin G/H synthase and cyclooxygenase)	1.924	0.000
ARTN	artemin	1.926	0.028
CPEB4	cytoplasmic polyadenylation element binding protein 4	1.927	0.005
LOC101927797	NA	1.939	0.020
WNT2B	wingless-type MMTV integration site family, member 2B	1.959	0.048
NAT1	N-acetyltransferase 1 (arylamine N-acetyltransferase)	1.971	0.039
DOC2A	double C2-like domains, alpha	2.001	0.040
UTP14C	UTP14, U3 small nucleolar ribonucleoprotein, homolog C (yeast)	2.006	0.015
IL1A	interleukin 1, alpha	2.027	0.049
TTLL7	tubulin tyrosine ligase-like family member 7	2.042	0.042
BMI1	BMI1 proto-oncogene, polycomb ring finger	2.049	0.001
NXPH4	neurexophilin 4	2.054	0.007
PPFIA4	protein tyrosine phosphatase, receptor type, f polypeptide (PTPRF), interacting protein (liprin), alpha 4	2.076	0.002
EGLN3	egl-9 family hypoxia-inducible factor 3	2.100	0.003
SLC25A27	solute carrier family 25, member 27	2.174	0.048
PFN1P2	profilin 1 pseudogene 2	2.181	0.040
LOX	lysyl oxidase	2.198	0.001
FER1L4	fer-1-like family member 4, pseudogene (functional)	2.262	0.011
CTNNA3	catenin (cadherin-associated protein), alpha 3	2.288	0.050
C10orf85	NA	2.323	0.020
TREM1	triggering receptor expressed on myeloid cells 1	2.324	0.031
RORA	RAR-related orphan receptor A	2.325	0.018
SMAD9	SMAD family member 9	2.343	0.044
AADAC	arylacetamide deacetylase	2.393	0.043
IPO9-AS1	IPO9 antisense RNA 1	2.457	0.042
FHIT	fragile histidine triad	2.487	0.040
CA9	carbonic anhydrase IX	2.489	0.000
TMEM27	transmembrane protein 27	2.491	0.037
ZNF474	zinc finger protein 474	2.492	0.011
AMY2B	amylase, alpha 2B (pancreatic)	2.528	0.031
SH3BP5-AS1	SH3BP5 antisense RNA 1	2.592	0.011
TICAM2	toll-like receptor adaptor molecule 2	2.607	0.012
ZNF415	zinc finger protein 415	2.640	0.021
LOC100507577	NA	2.657	0.020
FAM95B1	family with sequence similarity 95, member B1	2.721	0.033
LINC01024	long intergenic non-protein coding RNA 1024	2.808	0.009
CP	ceruloplasmin (ferroxidase)	2.922	0.000
KLHDC1	kelch domain containing 1	3.019	0.008

C2CD4A	C2 calcium-dependent domain containing 4A	3.056	0.022
CCDC180	coiled-coil domain containing 180	3.198	0.007
MPP7	membrane protein, palmitoylated 7 (MAGUK p55 subfamily member 7)	3.457	0.000
FKBP1A-SDCBP2	FKBP1A-SDCBP2 readthrough (NMD candidate)	3.493	0.041
JMJD7-PLA2G4B	JMJD7-PLA2G4B readthrough	3.856	0.040
EFCAB3	EF-hand calcium binding domain 3	4.110	0.008
NDUFA4L2	NADH dehydrogenase (ubiquinone) 1 alpha subcomplex, 4-like 2	4.450	0.000
LOC101928674	NA	4.528	0.008
ARMCX5-GPRASP2	ARMCX5-GPRASP2 readthrough	4.865	0.040
SHC4	SHC (Src homology 2 domain containing) family, member 4	5.287	0.003
GAGE12F	G antigen 12F	5.492	0.006
NEDD8-MDP1	NEDD8-MDP1 readthrough	156.391	0.000
C8orf44-SGK3	C8orf44-SGK3 readthrough	165.829	0.000
RAB4B-EGLN2	RAB4B-EGLN2 readthrough (NMD candidate)	287.408	0.000
EIF3CL	eukaryotic translation initiation factor 3, subunit C-like	821.897	0.000

Supplementary Table 8 – GSEA Enrichment Analysis investigating Hallmark Pathways in sorted lung samples GFP+ (LG) or DsRed+ (LR). Highlighted in green are the hallmarks with p-value≤0.05 and FDR≤0.25.

a) Enrichment in GFP+ lung metastatic cells (LG)

NAME	SIZE	ES	NES	NOM p-val	FDR q-val	FWER p-val	RANK AT MAX
HALLMARK_HYPOXIA	199	0.538715	1.798288	0	0.001766	0.002	4613
HALLMARK_PROTEIN_SECRETION	96	0.565594	1.701655	0	0.004375	0.01	7452
HALLMARK_TNFA_SIGNALING_VIA_NFKB	199	0.410184	1.362003	0.021978	0.260548	0.602	5456
HALLMARK_GLYCOLYSIS	199	0.410594	1.356235	0.01897	0.210373	0.626	4759
HALLMARK_ANDROGEN_RESPONSE	101	0.439238	1.351719	0.037559	0.176404	0.64	5046
HALLMARK_IL2_STAT5_SIGNALING	199	0.396644	1.319925	0.033333	0.207387	0.761	5016
HALLMARK_INFLAMMATORY_RESPONSE	198	0.360518	1.194521	0.110181	0.534351	0.993	4848
HALLMARK_UV_RESPONSE_DN	144	0.361415	1.156637	0.169118	0.634257	0.998	5333
HALLMARK_APOPTOSIS	160	0.324537	1.051924	0.335735	1	1	5657

HALLMARK_KRAS_SIGNALING_UP	199	0.302761	1.003585	0.462411	1	1	3649
HALLMARK_WNT_BETA_CATENIN_SIGNALING	42	0.358629	0.963757	0.503175	1	1	7342
HALLMARK_MTORC1_SIGNALING	199	0.28404	0.935021	0.651289	1	1	5232
HALLMARK_COMPLEMENT	196	0.282537	0.933023	0.625526	1	1	5366
HALLMARK_COAGULATION	136	0.294355	0.929393	0.62963	1	1	6010
HALLMARK_ADIPOGENESIS	196	0.275471	0.905878	0.719298	1	1	5890
HALLMARK_MYOGENESIS	200	0.270522	0.896173	0.740688	1	1	3419
HALLMARK_REACTIVE_OXIGEN_SPECIES_PATHWAY	47	0.325928	0.895098	0.660964	1	1	2951
HALLMARK_EPITHELIAL_MESENCHYMAL_TRANSITION	198	0.263402	0.878643	0.782313	1	1	4815
HALLMARK_UNFOLDED_PROTEIN_RESPONSE	112	0.283732	0.872582	0.768634	1	1	8297
HALLMARK_FATTY_ACID_METABOLISM	158	0.269902	0.870105	0.798246	1	1	5124
HALLMARK_ESTROGEN_RESPONSE_LATE	199	0.262624	0.868144	0.823695	1	1	3589
HALLMARK_IL6_JAK_STAT3_SIGNALING	87	0.284515	0.854919	0.76783	1	1	3302
HALLMARK_ESTROGEN_RESPONSE_EARLY	200	0.256448	0.842067	0.879214	1	1	5242
HALLMARK_CHOLESTEROL_HOMEOSTASIS	73	0.287363	0.833387	0.802198	1	1	3215
HALLMARK_PI3K_AKT_MTOR_SIGNALING	105	0.272798	0.833283	0.816964	1	1	7022
HALLMARK_ALLOGRAFT_REJECTION	200	0.249961	0.827914	0.914923	0.999125	1	4422
HALLMARK_MITOTIC_SPINDLE	198	0.25185	0.827646	0.912553	0.962548	1	8348
HALLMARK_HEME_METABOLISM	195	0.246006	0.824177	0.932584	0.936348	1	5867
HALLMARK_UV_RESPONSE_UP	156	0.237605	0.769222	0.976642	0.986962	1	4903
HALLMARK_TGF_BETA_SIGNALING	54	0.251257	0.696908	0.960191	0.989623	1	5232

b) Enrichment in DsRed+ lung metastatic cells (LR)

**NAME**	**SIZE**	**ES**	**NES**	**NOM p-val**	**FDR q-val**	**FWER p-val**	**RANK AT MAX**
HALLMARK_E2F_TARGETS	199	-0.46507	-1.67154	0	0.01911	0.014	5745
HALLMARK_G2M_CHECKPOINT	200	-0.357	-1.28632	0.01487	0.497579	0.56	5460

HALLMARK_PANCREAS_BETA_CELLS	40	-0.44942	-1.26127	0.127937	0.404974	0.631	2571
HALLMARK_MYC_TARGETS_V2	58	-0.41031	-1.20394	0.136364	0.47478	0.788	7100
HALLMARK_APICAL_SURFACE	44	-0.41786	-1.20334	0.160428	0.381676	0.79	2687
HALLMARK_OXIDATIVE_PHOSPHORYLATION	199	-0.31751	-1.15175	0.107266	0.484709	0.903	7217
HALLMARK_KRAS_SIGNALING_DN	195	-0.29674	-1.0645	0.281139	0.80057	0.995	4329
HALLMARK_DNA_REPAIR	143	-0.30047	-1.02991	0.350168	0.890122	0.999	6475
HALLMARK_APICAL_JUNCTION	200	-0.285	-1.01197	0.379928	0.891073	1	5719
HALLMARK_MYC_TARGETS_V1	199	-0.27758	-1.0113	0.363971	0.805322	1	6269
HALLMARK_SPERMATOGENESIS	133	-0.29065	-0.9914	0.454819	0.838448	1	3012
HALLMARK_INTERFERON_GAMMA_RESPONSE	197	-0.26995	-0.97753	0.537879	0.833004	1	4546
HALLMARK_INTERFERON_ALPHA_RESPONSE	94	-0.29346	-0.95618	0.565826	0.865878	1	5355
HALLMARK_XENOBIOTIC_METABOLISM	199	-0.25069	-0.89501	0.795918	1	1	4157
HALLMARK_BILE_ACID_METABOLISM	112	-0.24582	-0.83661	0.90991	1	1	5807
HALLMARK_HEDGEHOG_SIGNALING	36	-0.30795	-0.83589	0.773399	1	1	1672
HALLMARK_PEROXISOME	103	-0.25079	-0.82912	0.861027	1	1	5389
HALLMARK_P53_PATHWAY	199	-0.21967	-0.79109	1	1	1	4620
HALLMARK_ANGIOGENESIS	36	-0.27217	-0.7482	0.916667	1	1	2839
HALLMARK_NOTCH_SIGNALING	32	-0.23183	-0.62803	0.987715	0.998483	1	5460

Supplemental Table 9 – Results of VennPlex analysis overlapping gene expression (-1.5≥FC≥1.5, p-value≤0.05) in primary tumor (TG vs TR) with lung metastasis induction in culture conditions (LG vs LR).

LG/LR		TG/TR ∩ LG/LR		
Up-regulated Total = 102	*Down-regulated Total = 43*	***Up-regulated Total = 19***	*Contra-regulated Total = 2*	***Down-regulated Total = 2***
PKP2	LOC642423	**BMI1**	SSC5D	**GRM5-AS1**
ALDOC	LDLRAD2	**NDRG1**	GPC2	**HAS3**
TPD52	HNRNPA1P10	**RNASE4**		
KLF5	PLA2G4B	**CA9**		
RFK	LOC101928295	**FAM13A**		
HEATR5A	NCRUPAR	**CPEB4**		
F8	FLT4	**PTGS2**		
SOWAHC	SLC34A2	**PPFIA4**		
PSMD5-AS1	CGB	**IDI2**		
DBNDD2	FIRRE	**EGLN3**		
SBF2-AS1	ALPPL2	**DNAH11**		
IGFBP1	ZNF852	**WNT2B**		
AKAP12	ANPEP	**TTN**		
BNIP3L	FRMPD3	**LOX**		
L2HGDH	SCN11A	**RORA**		
ZMYM1	OLFML2B	**FER1L4**		
CXCR4	POM121L10P	**CP**		
GTF2H2	ZNF684	**JMJD7-PLA2G4B**		
BNIP3	HOMEZ	**C8orf44-SGK3**		
ADM	ZNF365			
DTWD1	PCDHGA3			
SLC36A4	FAM86JP			
MXI1	FGD3			
ZNF521	VIPR1			
CRIP2	LRRN4CL			
VLDLR	KCNJ12			
ZXDA	RAB37			
ZBTB21	PCDHGA12			
TMEM181	OXTR			
ERO1L	PDGFRB			
KIAA0232	RASL10A			
FAM45A	APOBEC3G			
MIR210HG	HCP5			
PDGFB	NCF2			
LRRC8B	ARHGAP5-AS1			

C10orf10	PAPLN			
ARSK	GDF15			
TP53INP1	PLCG2			
EML6	MYRF			
ZNF766	LAPTM5			
MTX3	SHROOM2			
SLC25A40	ADAMTS15			
WHAMMP1	TNFAIP8L1			
LIMCH1				
SLC35A3				
FKBP9P1				
POLK				
COX10-AS1				
PDE1C				
TPPP				
VGLL3				
SOCS4				
CCDC176				
RIMKLA				
GOLGA6L9				
HS3ST3B1				
IGFBP3				
NPIPB6				
CSGALNACT1				
BCL2A1				
SLIT2				
PRPF39				
ARTN				
LOC101927797				
NAT1				
DOC2A				
UTP14C				
IL1A				
TTLL7				
NXPH4				
SLC25A27				
PFN1P2				
CTNNA3				
C10orf85				
TREM1				
SMAD9				

AADAC				
IPO9-AS1				
FHIT				
TMEM27				
ZNF474				
AMY2B				
SH3BP5-AS1				
TICAM2				
ZNF415				
LOC100507577				
FAM95B1				
LINC01024				
KLHDC1				
C2CD4A				
CCDC180				
MPP7				
FKBP1A-SDCBP2				
EFCAB3				
NDUFA4L2				
LOC101928674				
ARMCX5-GPRASP2				
SHC4				
GAGE12F				
NEDD8-MDP1				
RAB4B-EGLN2				
EIF3CL				
