# Supplementary material for: Fate-mapping post-hypoxic tumor cells reveals a ROS-resistant phenotype that promotes metastasis
Source: Nat Commun. 2019 Oct 24;10:4862. doi: 10.1038/s41467-019-12412-1 (PMC6813355; doi:10.1038/s41467-019-12412-1)
Supplement: Supplementary file 3 — Description of Additional Supplementary Files [file 41467_2019_12412_MOESM3_ESM.docx]

**Description of Additional Supplementary Files**

**File Name: Supplementary Movie 1**

**Description:** MDA-MB-231 hypoxia fate-mapping cells under 0.5% O2. Hypoxia fate-mapping cells were incubated at 0.5% O2 and maintained at 37°C. Timelapse imaging was conducted at 10X every 2 h.

**File Name: Supplementary Movie 2**

**Description:** MDA-MB-231 hypoxia fate-mapping spheroid. 3D reconstruction of a spheroid derived of hypoxia fate-mapping MDA-MB-231 cells after 15 days in culture under 20% O2. 3D surface rendering is applied for the visualization of color distribution.

**File Name: Supplementary Movie 3**

**Description:** Hypoxia fate-mapping mouse derived organoid. Time lapse imaging of a large organoid derived from a triple-transgenic hypoxia fate-mapping mouse cultured under 20% O2 over a 10-day time period.
