# Supplementary material for: Fate-mapping post-hypoxic tumor cells reveals a ROS-resistant phenotype that promotes metastasis
Source: Nat Commun. 2019 Oct 24;10:4862. doi: 10.1038/s41467-019-12412-1 (PMC6813355; doi:10.1038/s41467-019-12412-1)
Supplement: Supplementary file 7 — Reporting Summary [file 41467_2019_12412_MOESM7_ESM.pdf]

nature research

Corresponding author(s): Daniele M. Gilkes, PhD

Last updated by author(s): Aug 16, 2019

Reporting Summary

Nature Research wishes to improve the reproducibility of the work that we publish. This form provides structure for consistency and transparency in reporting. For further information on Nature Research policies, see Authors & Referees and the Editorial Policy Checklist.

Statistics

For all statistical analyses, confirm that the following items are present in the figure legend, table legend, main text, or Methods section.

n/a	Confirmed	
☐	☒	The exact sample size (n) for each experimental group/condition, given as a discrete number and unit of measurement
☐	☒	A statement on whether measurements were taken from distinct samples or whether the same sample was measured repeatedly
☐	☒	The statistical test(s) used AND whether they are one- or two-sided<br>*Only common tests should be described solely by name; describe more complex techniques in the Methods section.*
☐	☒	A description of all covariates tested
☐	☒	A description of any assumptions or corrections, such as tests of normality and adjustment for multiple comparisons
☐	☒	A full description of the statistical parameters including central tendency (e.g. means) or other basic estimates (e.g. regression coefficient) AND variation (e.g. standard deviation) or associated estimates of uncertainty (e.g. confidence intervals)
☐	☒	For null hypothesis testing, the test statistic (e.g. F, t, r) with confidence intervals, effect sizes, degrees of freedom and P value noted<br>*Give P values as exact values whenever suitable.*
☒	☐	For Bayesian analysis, information on the choice of priors and Markov chain Monte Carlo settings
☒	☐	For hierarchical and complex designs, identification of the appropriate level for tests and full reporting of outcomes
☐	☒	Estimates of effect sizes (e.g. Cohen's d, Pearson's r), indicating how they were calculated

Our web collection on statistics for biologists contains articles on many of the points above.

Software and code

Policy information about availability of computer code

Data collection	Not applicable.
Data analysis	Not applicable.

For manuscripts utilizing custom algorithms or software that are central to the research but not yet described in published literature, software must be made available to editors/reviewers. We strongly encourage code deposition in a community repository (e.g. GitHub). See the Nature Research guidelines for submitting code & software for further information.

Data

Policy information about availability of data

All manuscripts must include a data availability statement. This statement should provide the following information, where applicable:

- Accession codes, unique identifiers, or web links for publicly available datasets
- A list of figures that have associated raw data
- A description of any restrictions on data availability

The RNA sequencing data have been deposited in the GEO database under the accession codes GSE111653 (in vitro hypoxia) [https://www.ncbi.nlm.nih.gov/geo/query/acc.cgi?acc=GSE111653], GSE126609 (tumor) [https://www.ncbi.nlm.nih.gov/geo/query/acc.cgi?acc=GSE126609] and GSEXXXX (lung). The survival data referenced during the study are available in a public repository from the kmplot.com website. All the other data supporting the findings of this study are available within the article and its supplementary information files and from the corresponding author upon reasonable request. A reporting summary for this article is available as a Supplementary Information file.

Field-specific reporting

Please select the one below that is the best fit for your research. If you are not sure, read the appropriate sections before making your selection.

☒ Life sciences ☐ Behavioural & social sciences ☐ Ecological, evolutionary & environmental sciences

For a reference copy of the document with all sections, see nature.com/documents/nr-reporting-summary-flat.pdf

Life sciences study design

All studies must disclose on these points even when the disclosure is negative.

Sample size	Samples size for studies in 3D in vitro models consisted of three (N=3) biological repeats, except in MCF7 spheroids (N=2) with large number of technical repeats (n=16-27 spheroids and n>200 organoids).<br>Animal studies in the orthotopic MDA-MB-231 model included a total of 48 mice to investigate tumor progression across three biological repeats (N=3, n=9, n=27, n=12). Oxygen was measured in 8 mice in one of the biological repeats (N=1, n=8). GFP+ micrometastasis were observed in 23 mice and macrometastasis were observed in 26 mice across three biological repeats (micro: N=2, n=14, n=9 and macro: N=3, n=3, n=14, n=9). Tail vein injection was performed in a set of 5 mice (N=1, n=5). Sample size of the other animal studies: 4T1 orthotopic model (N=2, n=5), MCF7 intraductal model (N=1, n=5) and triple-transgenic model (N=10).<br>RT-PCR was performed in three biological repeats with three technical repeats (N=3, n=3), except for the supplementary reoxygenation in tumor derived cells (N=2) and in the cells cultured in vitro study (N=1).<br>Western blotting, flow cytometry and live cell time-lapse were performed one to three times, since different methods provided consistent results.
Data exclusions	About 5-10% of the MDA-MB-231 spheroids were excluded due to damaged structure caused by the complexity of the model. Only rounded and intact spheroids were considered.
Replication	All experiments were reproducible. Biological repeats were performed to confirm consistency of the observations.
Randomization	Not applicable.
Blinding	Not applicable.

Reporting for specific materials, systems and methods

We require information from authors about some types of materials, experimental systems and methods used in many studies. Here, indicate whether each material, system or method listed is relevant to your study. If you are not sure if a list item applies to your research, read the appropriate section before selecting a response.

Materials & experimental systems

n/a	Involved in the study
☐	☒ Antibodies
☐	☒ Eukaryotic cell lines
☒	☐ Palaeontology
☐	☒ Animals and other organisms
☒	☐ Human research participants
☒	☐ Clinical data

Methods

n/a	Involved in the study
☒	☐ ChIP-seq
☐	☒ Flow cytometry
☒	☐ MRI-based neuroimaging

Antibodies

Antibodies used	HIF-1α (BD Biosciences 610959), GFP (Abcam 13970), β-actin HRP-conjugated (Proteintech HRP-60008), HIF-1α (Santa Cruz Biotechnology SC-10790), Hypoxyprobe™-1, and CD31 (Abcam 28364).
Validation	HIF-1α (BD Biosciences 610959) has been previously validated by our work in WB by staining induction upon 1% O2 exposure when compared to 20% O2, and it was further confirmed in HIF-KO studies (Ju et al. 2017).<br>GFP (Abcam 6556) specificity was validated in WB by using a GFP overexpressed cell line (GFP+/DsRed-) versus a RFP overexpressed cell line (GFP-/DsRed+).<br>HIF-1α (Santa Cruz Biotechnology SC-10790) and Hypoxyprobe™-1 were validated in IF staining since they overlapped and lined the necrotic core of the tumor (confirmed by Tunel staining) as expected. Moreover, Hypoxyprobe™-1 staining has been extensively validated by many labs and also in our previous work (Belcher et al. 2018) by comparing mouse tumors with and without pimonidazole injection. HIF-1α (Santa Cruz Biotechnology SC-10790) has also been validated in our previous work (Ju et al. 2017).<br>CD31 (Abcam 28364) is widely used to mark blood vessels (citations > 800) and we have also reported it before (Belcher et al. 2018).

Eukaryotic cell lines

Policy information about cell lines

Cell line source(s)	MDA-MB-231, MCF7 and 4T1 cells were obtained from the American Type Culture Collection (ATCC).
Authentication	Cell lines were authenticated in the Genetic Resources Core Facility at the Johns Hopkins School of Medicine by short tandem repeat (STR) profiling and comparison to a known profile.
Mycoplasma contamination	Mycoplama contamination testing was performed by the Genetic Resources Core Facility at the Johns Hopkins School of Medicine using a PCR based MycoDtect kit from Greiner Bio-Onein in 2017. We test our cells every 3 months in the lab using the PCR based Angilent kit #302106. All cell lines tested negative.
Commonly misidentified lines (See ICLAC register)	MCF7.

Animals and other organisms

Policy information about studies involving animals; ARRIVE guidelines recommended for reporting animal research

Laboratory animals	Female 5- to 7-week-old NOD-SCID Gamma (NSG) or BALB/c (Charles River Laboratories) were used in our studies, except in the intraductal mouse model, where 8 to 12-month-old multiparous (NSG) female mice were required.
Wild animals	Not applicable.
Field-collected samples	Not applicable.
Ethics oversight	Protocols were approved by the Johns Hopkins University Animal Care and Use Committee.

Note that full information on the approval of the study protocol must also be provided in the manuscript.

Flow Cytometry

Plots

Confirm that:

☒ The axis labels state the marker and fluorochrome used (e.g. CD4-FITC).

☒ The axis scales are clearly visible. Include numbers along axes only for bottom left plot of group (a 'group' is an analysis of identical markers).

☒ All plots are contour plots with outliers or pseudocolor plots.

☒ A numerical value for number of cells or percentage (with statistics) is provided.

Methodology

Sample preparation	Adherent cells in 2D cultures were trypsinized, resuspended in culture media containing FBS, washed in PBS and collected in sorting buffer (PBS, 1% BSA, 0.5 mM EDTA, 25 mM HEPES pH 8). Organoids' gels were incubated with trypsin for 10 min, washed twice with PBS, collected in FACS buffer and strained into the FACS tube.<br>Tumor-derived cell lines were generated from tumors harvested 2 weeks after implantation. Tumors were harvested and loose tissue was carefully removed. Tumors were then sectioned in half and the necrotic core was carefully detached. With a razor blade, tumors were manually chopped into fine fragments and transferred to a digestion mix (collagenase 2 mg/mL (Sigma-Aldrich), BSA 2 mg/mL (Gemini Bioproducts)). The suspension was incubated for 1 h at 37°C at 160 RPM in an orbital shaker. The suspension was then filtered (0.70 µm strainer) and washed twice with PBS. Single cells of human origin were enriched by magnetic-activated cell separation (MACS) using a Mouse Cell Depletion Kit (Miltenyi Biotec) following the manufacturer's instructions. The resultant cell suspension was then washed with PBS and suspended in sorting buffer.
Instrument	FACSAria II (BD Biosciences) and SH800 (Sony Biotechnology).
Software	Data was collected with FACSDiva (BD Biosciences) or SH800 software (Sony Biotechnology). Data was analysed on FlowJo V10.
Cell population abundance	MDA-MB-231, MCF7 and 4T1 hypoxia fate-mapping cells were sorted for enrichment in terms of fluorescence intensity. Post-sort tests indicated 95-100% purity in terms of DsRed/(DsRed+GFP). Tumor MDA-MB-231 samples were sorted for RNA or to generate cells lines. Post-sort tests indicated 90-100% purity in terms of DsRed/(DsRed+GFP), and 100% purity in terms of fluorescently labeled cells/non-labeled cells.
Gating strategy	FSC and SSC were defined in order to display the entire cell population and were used consistently throughout the work. Doublet-discrimination was used after FSC/SSC gating using FSC-H/FSC-A. DsRed and GFP were compensated using DsRed-/GFP-, DsRed+/GFP- and DsRed-/GFP+ controls. Similar strategy was used in the organoids studies.

☒ Tick this box to confirm that a figure exemplifying the gating strategy is provided in the Supplementary Information.
